# Projecting the 10-year costs of care and mortality burden of depression until 2032: a Markov modelling study developed from real-world data

**DOI:** 10.1016/j.lanwpc.2024.101026

**Published:** 2024-02-06

**Authors:** Vivien Kin Yi Chan, Man Yee Mallory Leung, Sandra Sau Man Chan, Deliang Yang, Martin Knapp, Hao Luo, Dawn Craig, Yingyao Chen, David Makram Bishai, Gloria Hoi Yan Wong, Terry Yat Sang Lum, Esther Wai Yin Chan, Ian Chi Kei Wong, Xue Li

**Affiliations:** a Department of Pharmacology and Pharmacy, Li Ka Shing Faculty of Medicine, The University of Hong Kong, Hong Kong SAR, China; b Department of Medicine, School of Clinical Medicine, Li Ka Shing Faculty of Medicine, The University of Hong Kong, Hong Kong SAR, China; c Faculty of Business and Economics, The University of Hong Kong, Hong Kong SAR, China; d Department of Psychiatry, Faculty of Medicine, The Chinese University of Hong Kong, Hong Kong SAR, China; e Department of Health Policy, London School of Economics and Political Science, United Kingdom; f Department of Social Work and Social Administration, Faculty of Social Sciences, The University of Hong Kong, Hong Kong SAR, China; g Population Health Sciences Institute, Faculty of Medical Sciences, Newcastle University, United Kingdom; h National Health Commission Key Laboratory of Health Technology Assessment, Fudan University, China; i Division of Health Economics, Policy and Management, School of Public Health, Li Ka Shing Faculty of Medicine, The University of Hong Kong, Hong Kong SAR, China; j Laboratory of Data Discovery for Health (D^2^4H), Hong Kong SAR, China; k Research Department of Policy and Practice, University College London School of Pharmacy, London, United Kingdom

**Keywords:** Burden projection, Cost, Mortality, Depression, Treatment-resistant depression, Comorbidities, Health policy, Real-world data, Time-varying Markov model, Real-world evidence

## Abstract

**Background:**

Based on real-world data, we developed a 10-year prediction model to estimate the burden among patients with depression from the public healthcare system payer's perspective to inform early resource planning in Hong Kong.

**Methods:**

We developed a Markov cohort model with yearly cycles specifically capturing the pathway of treatment-resistant depression (TRD) and comorbidity development along the disease course. Projected from 2023 to 2032, primary outcomes included costs of all-cause and psychiatric care, and secondary outcomes were all-cause deaths, years of life lived, and quality-adjusted life-years. Using the territory-wide electronic medical records, we identified 25,190 patients aged ≥10 years with newly diagnosed depression from 2014 to 2016 with follow-up until 2020 to observe the real-world time-to-event pattern, based on which costs and time-varying transition inputs were derived using negative binomial modelling and parametric survival analysis. We applied the model as both closed cohort, which studied a fixed cohort of incident patients in 2023, and open cohort, which introduced incident patients by year from 2014 to 2032. Utilities and annual new patients were from published sources.

**Findings:**

With 9217 new patients in 2023, our closed cohort model projected the 10-year cumulative costs of all-cause and psychiatric care to reach US$309.0 million and US$58.3 million, respectively, with 899 deaths (case fatality rate: 9.8%) by 2032. In our open cohort model, 55,849–57,896 active prevalent cases would cost more than US$322.3 million and US$60.7 million, respectively, with more than 943 deaths annually from 2023 to 2032. Fewer than 20% of cases would live with TRD or comorbidities but contribute 31–54% of the costs. The greatest collective burden would occur in women aged above 40, but men aged above 65 and below 25 with medical history would have the highest costs per patient-year. The key cost drivers were relevant to the early disease stages.

**Interpretation:**

A limited proportion of patients would develop TRD and comorbidities but contribute to a high proportion of costs, which necessitates appropriate attention and resource allocation. Our projection also demonstrates the application of real-world data to model long-term costs and mortality, which aid policymakers anticipate foreseeable burden and undertake budget planning to prepare for the care need in alternative scenarios.

**Funding:**

Research Impact Fund from the University Grants Committee, Research Grants Council with matching fund from the Hong Kong Association of Pharmaceutical Industry (R7007-22).

**Research in context**

**Evidence before this study**

We searched PubMed and Google Scholar for research articles up to 16th August, 2023 in English and Chinese using the terms (project∗ OR predict∗ OR forecast∗) AND model∗ AND depress∗ AND (burden OR economic OR cost∗ OR mortality) and found two relevant models studying the burden of depression. One regression-based model in 2006 focused on the global burden of mortality and disabilities until 2030 due to injuries, acute and chronic conditions and projected that depression would rank the top three causes of disability-adjusted life-years across 98 countries. Another simulation-based model in the United Kingdom projected rising future prevalence, service cost and productivity loss due to depression from 2007 until 2026 alongside seven types of other mental health conditions. However, the models were designed more than a decade ago and did not fully consider the development of treatment resistance and comorbidities, which are two key states that further intensify the burden of mortality and costs of care during the course of depression. Other less relevant published models were also designed for evaluating depression treatments using trial data, which model structures were less adaptive to studying care burden in a long-term and real-world setting.

**Added value of this study**

To our knowledge, we present the first projection model that accounted for the trajectories linking treatment-resistant depression (TRD) and new-onset comorbidities based on real-world data to reflect their roles on disease and economic burden in the disease course. We provided descriptive estimates of the 10-year cumulative and annual costs of all-cause and psychiatric care and deaths until 2032 in both closed and open cohort scenarios, considering alternative scenarios brought by the impact of the COVID-19 pandemic. Despite a limited proportion of cases living with TRD or comorbidities, they were projected to account for a substantial proportion of costs. Most of the key cost determinants were relevant to the early stages of the disease. Our model also suggested that the greatest collective burden occurred in the middle-aged and older women, but the older men and adolescent men with medical history had the highest individual costs instead.

**Implications of all the available evidence**

Global evidence suggests significant non-fatal and fatal burden associated with depression. Our projection model provides policymakers an understanding of resource allocation in the real-world setting and insights to early budget planning for the preparedness of the growing need in mental health services. Policymakers should be alerted that preventive interventions and treatments targeting the early stages of disease could lead to the greatest cost-savings, particularly before the occurrence of substantial burden once patients proceed to TRD or comorbidities. With the flexible modelling study design and open-sourced programming, the model could also be applied to estimate the changes in patient journey and magnitude of cost-savings by setting hypothetical scenarios specific to incidence of depression, TRD and/or comorbidities for further resource planning from various jurisdictions.

## Introduction

Depression is a debilitating mental health disorder affecting 280 million people worldwide.^1^ The World Health Organisation (WHO) estimates that depression accounts for 4.3% of the global disease burden and will rank as the leading contributor by 2030 due to premature deaths and years living with disabilities.^2^ Women and men suffering from depression have their life expectancies reduced by seven and ten years respectively.^3^, ^4^, ^5^ The chronicity and recurrent nature of the disease also lead to ongoing treatment needs and long-term costs. A recently published meta-analysis revealed that adults with depression had 2.6-fold and greater healthcare expenditures and 2.3-fold greater productivity losses than adults without depression.^6^ The situation is unlikely to improve, rather in the last decade, there have been rising trends in incidence and costs of depression across countries in Asia, Europe, and the United States (U.S.),^7^, ^8^, ^9^, ^10^, ^11^ highlighting that the immense burden is likely to persist, if not increase, in the near term.

Despite the availability of treatment and care, over half of patients are non-responsive to their initial antidepressants, and a notable proportion fail to reach remission even with subsequent regimens.^12^ This condition is known as treatment-resistant depression (TRD). In Hong Kong, patients with TRD have 1.5-fold greater risk of all-cause mortality and 1.8-fold higher cost of care in both psychiatric and non-psychiatric resources, compared with treatment responders.^13^ Depression is linked with subsequent onset of comorbidities, ranging from autoimmune diseases, cardiovascular disease, psychosis to anxiety disorder.^14^, ^15^, ^16^, ^17^, ^18^ The risks of developing these comorbidities also tend to increase further in the presence of TRD.^19^^,^^20^ An earlier study also found that the development of post-TRD comorbidities could mediate heightened mortality risk in patients with TRD.^13^ Consequently, the development of treatment resistance and comorbidities along the disease course could further intensify the risk of mortality and cost burdens in depression. However, most published economic models to date have not accounted for these features, due in part to their purposes in evaluating treatments instead of care burden projection, and the reliance on trial data in modelling short-term consequences.

Mental health has been recognised as a priority in Hong Kong. From 2014 to 2019, the government increased its expenditure on mental health services by 32% with the aim of enhancing workforce and inpatient resources.^21^ In 2017, the Advisory Committee on Mental Health emphasised the need to establish a long-term mental health policy with concrete resources and timetables to address future care needs.^21^ In response, we have developed an economic model to 1) project the 10-year costs of care and mortality from 2023 to 2032 and 2) identify the key drivers for cost-savings in care services for patients with depression. Our model accounted for the trajectories linking TRD and new-onset comorbidities using real-world data to reflect their impacts on cost and mortality. We anticipate that our findings would assist policymakers in early resource planning for the growing needs of overall mental health services.

## Methods

### Overview

We employed a decision-analytic modelling approach. Our primary outcomes were costs in all-cause and psychiatric-related care, and secondary outcomes included all-cause deaths, years of life lived, and quality-adjusted life-years (QALYs). Annual and cumulative estimates were produced. The model had a 10-year horizon (equivalent to two terms of government leadership) from the payer perspective in the public healthcare setting in Hong Kong. We used one-year cycle length, as it was considered appropriate to capture new prescriptions and diagnoses during the disease course.^22^^,^^23^ Costs are reported in 2023 US Dollars (US$) at an exchange rate of 7.85 Hong Kong Dollars.

### Integration of real-world data

In contrast to trial data, the use of real-world data enables the modelling of TRD and comorbidities pathways in the long-term setting and betters generalises the modelled outcomes to the local economic and clinical context. We prioritised the source of patient characteristics and model inputs to our territory-wide electronic medical records (EMR) database, supplemented by literature review when local data were absent. The data source was the Clinical Data Analysis and Reporting System managed by the Hospital Authority, an organisation managing all publicly funded healthcare services to eligible residents (>7.4 million) and covers 73% of hospital admissions. Patient data including demographics, deaths, attendances and diagnoses recorded in International Classification of Diseases, 9th Revision, Clinical Modification (ICD-9-CM) codes and prescriptions were recorded across all service settings for research and auditing purposes. The database has been widely used in depression-related epidemiological studies^13^^,^^15^^,^^19^ with reliable and validated diagnostic codes.^24^^,^^25^ In this study, we identified a cohort of patients aged above 10 years with newly diagnosed depression (ICD-9-CM: 296.2, 300.4, 311) between 2014 and 2016 from the database (the “2014–2016 cohorts” thereafter). New diagnosis was ascertained by reviewing diagnosis records since 1993, when the database was first available. Incident cohorts were followed up for a maximum of seven years to observe their real-world patterns of drug use, new diagnoses, service attendance and deaths until December 2020 to derive and validate input parameters. Actual number of newly diagnosed depression cases from 2014 to 2018 were also recorded. Parameter derivation was performed using R 4.0.3 and model simulation was cross-validated using Microsoft Excel and Python platforms by independent investigators (VC & DL).

### Model description

**Fig. 1:**
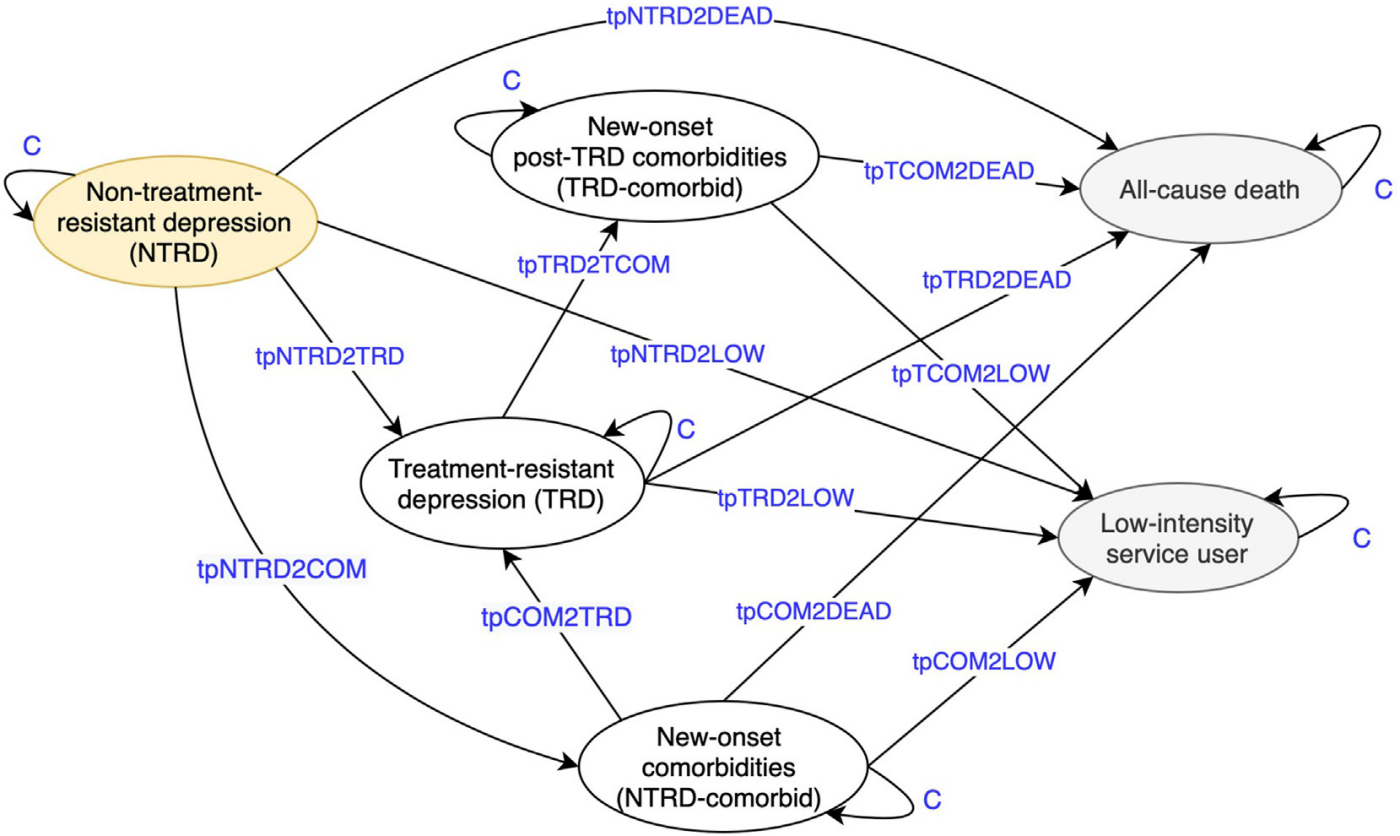
Schematic presentation of the Markov model structure. Yellow oval represents the initial state, grey ovals represent the absorbing states and blue texts represent the time-varying transition probabilities. “C” represents the complement of other probabilities from the same state. The definitions of the health states are as follows. *Non-treatment-resistant depression (NTRD)*: Patients with depression who were yet to develop TRD or further clinical characteristics. *Treatment-resistant depression (TRD)*: Patients who took at least two antidepressant regimens for an adequate duration and had the third regimen to confirm refractoriness in the first two regimens. *New-onset comorbidities (NTRD-comorbid)*: Patients with new-onset somatic comorbidities included in the list of diseases used to calculate Charlson Comorbidity Index, or pre-specified psychiatric comorbidities before TRD, and the new-onset condition(s) did not occur before depression diagnosis. *New-onset post-TRD comorbidities (TRD-comorbid)*: Similar to *NTRD-comorbid* but condition(s) occurred only after TRD. *Low-intensity service user (absorbing state)*: Patients with minimal care need and free of further depression-related diagnosis records and antidepressant prescriptions. The health state acts as a proxy for recovery from depression, rather than relapse or recurrence manifested during the development of TRD. Patients in this state are not considered as active or living with depression. *All-cause death (absorbing state)*: Observable deaths regardless of causes.

We chose a cohort Markov model due to its wide usage in decision-making and suitability for chronic disease modelling (Fig. 1).^26^^,^^27^ The model consisted of six health states: non-treatment-resistant depression (NTRD), treatment-resistant depression (TRD), new-onset comorbidities among NTRD patients (NTRD-comorbid), new-onset post-TRD comorbidities among TRD patients (TRD-comorbid), low-intensity service users and all-cause deaths. Detailed definitions are listed in Supplementary Table S1. To relax the Markovian ‘memoryless’ assumption, separate comorbidity states were introduced before and after TRD reflecting the different transition probabilities between states due to different disease stages. All trajectories and state definitions were supported by local expert opinion (SC) and treatment guidelines, therefore reflecting actual clinical practice.^28^ Our population of interest was patients aged above 10 years newly diagnosed with depression in the public hospitals from 2014 to 2032 (19 incident cohorts in total based on year of diagnosis), assuming their patient characteristics (*i.e.,* age, sex, and baseline medical history) were similar to those of the 2014–2016 cohorts referenced from the EMR database. The model assumed all patients started in the non-TRD state before transiting to the rest of natural disease course. Although discounting was not applied to main results to allow for visualisation of the original costing trend, discounted outcomes at the rate of 2.5% are presented in Supplementary Materials.^29^

### Model input parameters

All input parameters and respective distributions are listed in Supplementary Tables S2A–C. The sections below outline the principles of parameter derivation whilst the detailed methods are described in Supplementary Methods.

#### Time-varying transition probabilities

In chronic disease modelling, transition probabilities vary over the prolonged disease course. Our exploratory analysis showed that the crude annual probabilities of developing TRD from the non-TRD state to decline over time (1st year: 6.2%, 2nd year: 4.4%, 3rd year: 3.0%). This could be because, as cycles elapse, those who remained in the risk pool as non-treatment-resistant patients were likely to exhibit long-term stability. We thus adopted time-varying transitions in the model (Supplementary Table S2C). We performed parametric survival modelling for all transitions, censoring at health states or December 2020 with adjustment for age, sex, and baseline medical history. The definitions of medical history are listed in Supplementary Table S3. We fitted five standard distributions of the outcome (exponential, Weibull, lognormal, log-logistic and Gompertz), selecting the best-fit based on lowest values of Akaike information criterion (AIC) and Bayesian information criterion (BIC) (Supplementary Table S4) alongside visual inspection of the fitted curves against Kaplan–Meier plots.^30^, ^31^, ^32^ At yearly cycle *t*, probabilities were derived by P(t) = S(t)/S (t-1) where S(t) refers to the survival function.^27^^,^^30^ These estimates were validated by comparing modelled cumulative deaths and low-intensity service users at cycle 4 to those observed in the real-world cohorts at the fourth year. The mean absolute percentage errors ranged from 0.6% to 2.66%, indicating a highly accurate forecast^33^^,^^34^ (Supplementary Tables S5–S7).

#### Costs of care

The detailed methods to derive the costs of care per state were described in a previous cohort study.^13^ Costs related to healthcare service use were estimated by multiplying service-specific unit costs by frequency of using each service. Unit costs were taken from the non-subsidised charges released by the Hospital Authority in 2023 (Supplementary Table S8). Based on 2014–2016 reference incident cohorts with follow-up until the December 2020, frequency of visits was analysed as all-cause and psychiatric-related visits or bed-days in the outpatient, inpatient and emergency settings. We then fitted negative binomial regression models and adjusted for the matched variables (age, sex, and baseline medical history), TRD and comorbidities onset status during the follow-up to obtain costs of care per state stratified by subgroups. Costs related to low-intensity service user and all-cause death states were assumed to be not depression-related by definition (Supplementary Table S2A).

#### Utilities

We obtained utility weight per state based on literature review of the health-related quality-of-life (HRQoL) of patients with TRD and comorbidities using pre-set ranking criteria (Supplementary Table S9). Given the scant HRQoL literature on the two comorbidity states related to treatment resistance (NTRD-comorbid and TRD-comorbid), we assumed that the utilities were 17% lower than those of the non-comorbid states of NTRD and TRD.^35^, ^36^, ^37^, ^38^, ^39^ All selected articles passed our quality assessment satisfactorily (Supplementary Tables S10A–C).

#### Number of newly diagnosed patients

We analysed annual number of newly diagnosed cases of depression between 2014 and 2018 and the five-year mean incidence of newly diagnosed patients. We assumed incidence to be constant and projected the number of new patients in 2023 by multiplying mean age-specific annual incidence by the official population projections of each five-year age group released by the government.^40^ Although the WHO ended the global health emergency due to COVID-19 in May 2023,^41^ slow economic recovery and negative impact on mental health might persist.^42^ We thus estimated two further incidence profiles for 2023 by assuming 1) no extra new cases due to the pandemic from 2023 onwards, and 2) extra new cases (+25%) in 2023 due to the pandemic which resolved from 2024 onwards in both the base-case and scenario analyses. The derivation of extra numbers of cases came from the findings of a previous large-scale local prospective cohort study^43^ (Supplementary Table S2B). Depression is often undiagnosed in Hong Kong with only approximately half of individuals with probable depression seeking professional help, we recognise that our projected burden could represent a conservative underestimate.

### Analyses

#### Base-case and subgroup analyses

Our base-case scenario was a closed cohort, where we restricted the population to one reference incident cohort. We used only patients newly diagnosed in 2023 without adding new patients to reflect the ensuing 10-year trend of burden in accordance with the natural disease course. In the base-case scenario, we stratified outcomes into 16 subgroups by age group, sex, and medical history and compared their differences in the cumulative 10-year burden and the annualised per-patient burden. Also, as age and sex distribution could be different over the years, we additionally tested if applying the demographics characteristics observed among the real-world patients of 2018 would change the projected estimates, in contrast to assuming patients to follow the age and sex structure of the reference cohort diagnosed in 2014–2016 in the base case.

#### Scenario analysis

We additionally projected outcomes in an open cohort scenario, where we introduced new incident cohorts into the model on a yearly basis. Different from the closed cohort setting, this scenario reflects actual burden in any current year from 2023 to 2032 among prevalent patients, which is more relevant to direct resource allocation. We introduced the first cohort into the model from 2014, i.e., ten years before 2023, and simulated until 2032 with 19 years of cohorts in total. All 19 cohorts were simulated for a maximum of 10 cycles, thus included patients in any given calendar year refer to only patients newly diagnosed in the previous 10 years (“prevalent cases” thereafter) (Supplementary Figure S1). Derivation of numbers of new patients from 2019 to 2032 (model input) was similar to that in the closed cohort scenario but considered the extra new cases (+33%) due to the social movement in 2019 and the pandemic from 2020 to 2022^43^^,^^44^ (Supplementary Table S2B).

#### Sensitivity analyses

We assessed the robustness of the base-case scenario with deterministic sensitivity analysis (DSA) and probabilistic sensitivity analysis (PSA) on primary outcomes. In the DSA, we varied each parameter to its lower and upper bounds with the other parameters kept constant. This also helps identify key determinants for all-cause and psychiatric costs of care. Ranges of uncertainties were expressed in ±95% confidence intervals (CI) of the base-case values, or a 20% variation if CIs were not available. In the PSA, we present mean projected costs by varying all model inputs simultaneously under the assigned distributions (Supplementary Tables S2A–C) with 1000 Monte Carlo iterations. The codes and model are available on Dataverse and GitHub via https://doi.org/10.7910/DVN/ZVBFVA and https://github.com/scan2030.

### Ethics approval

This study received ethics approval from the Institutional Review Board of The University of Hong Kong/Hospital Authority Hong Kong Western Cluster (UW 20-218).

### Role of the funding source

This study is supported by the Research Impact Fund from the University Grants Committee, Research Grants Council, and Matching Funds from the Hong Kong Association of Pharmaceutical Industry. The funding source has no role in the study design, in the collection, analysis and interpretation of data, in the writing of manuscript and in the decision to submit the paper for publication.

## Results

The baseline characteristics of the 2014–2016 reference cohort for parameter derivation are shown in Supplementary Table S11.

### Base-case analysis (closed cohort model)

**Fig. 2:**
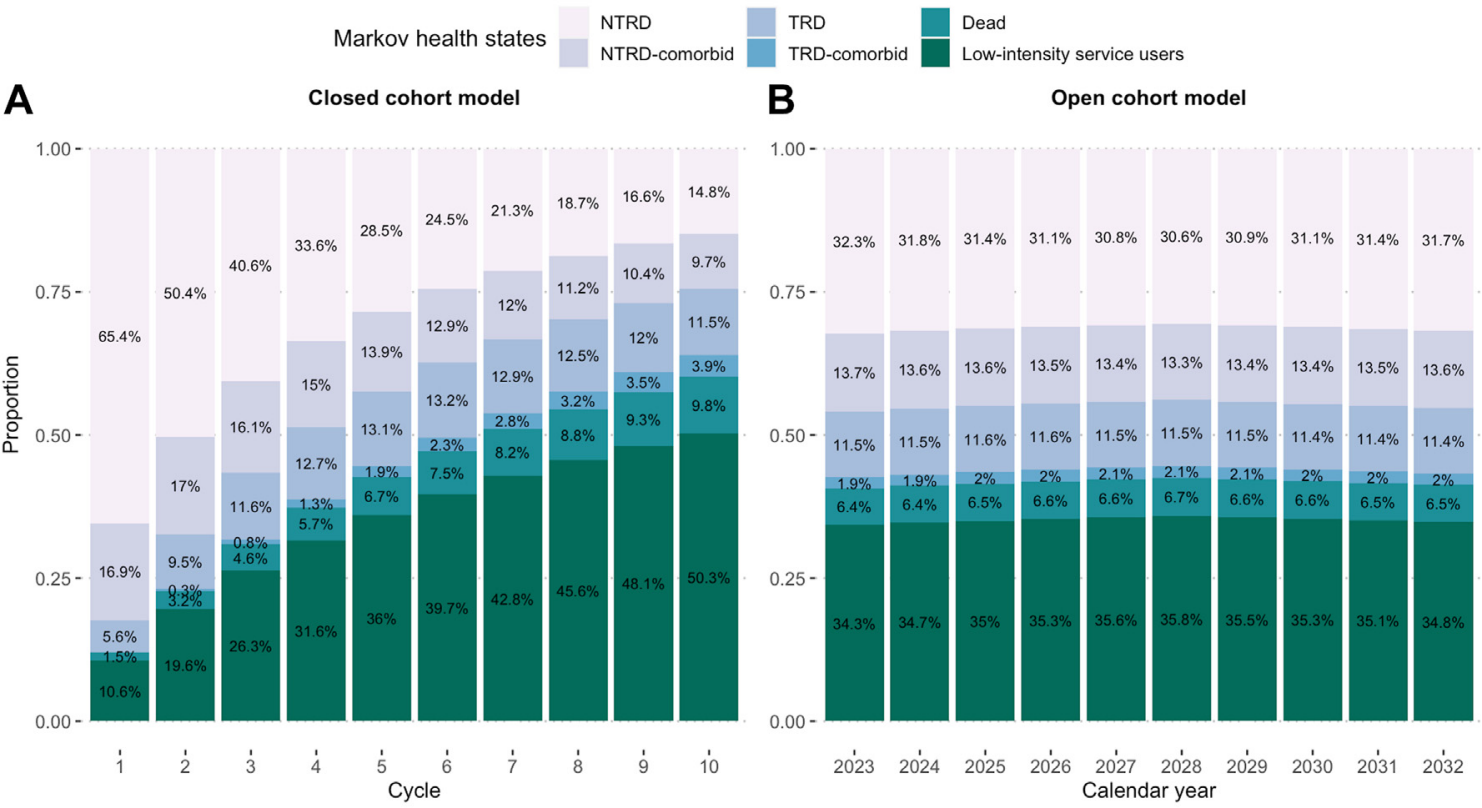
Modelled trajectories of 10-year patient distribution and flow between health states. All health states are mutually exclusive. The closed cohort model shows the 10-year flow among the incident patients diagnosed in 2023, with the tenth cycle equivalent to the year 2032. The open cohort model shows the annual snapshot of patient distribution between states among the patients diagnosed in the recent 10 years counting from the corresponding calendar year. The pandemic impact was not accounted. Abbreviations: NTRD, Non-treatment-resistant depression; TRD, Treatment-resistant depression.

In 2023, the estimated numbers of patients with newly diagnosed depression were 9147 (15.0 per 10,000 population) without residual pandemic impact. By the tenth cycle, we estimated that 15.4% and 13.6% of patients would be living in TRD states or new-onset comorbidities states (Fig. 2A). Among active patients living with depression, consistent with our 2014–2016 reference cohorts, most were women (74.8%) diagnosed between ages 41 and 65 (55.6%) without baseline medical history (71.9%) by the tenth cycle.

#### Cumulative and annual costs of care

**Fig. 3:**
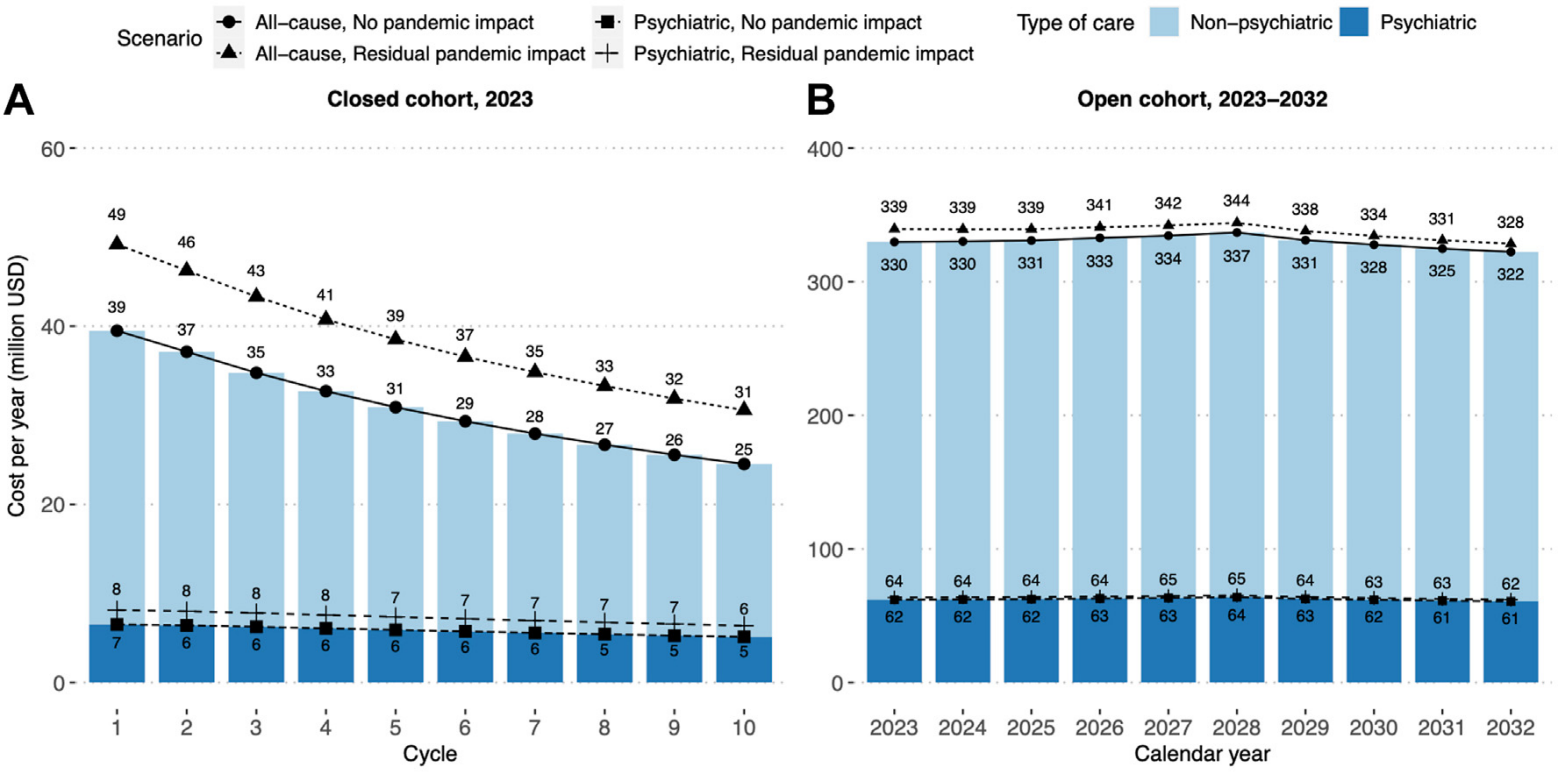
Projected annual costs of all-cause and psychiatric care from 2023 to 2032. All costs are undiscounted and valued in 2023 U.S. Dollars (USD). The closed cohort setting shows the projected annual costs of care among the incident patients diagnosed in 2023, with the tenth cycle equivalent to the year 2032. The open cohort setting shows the projected annual costs of care among the patients diagnosed in the recent 10 years counting from the corresponding calendar year.

Cumulative undiscounted cost of all-cause care for the 2023 incident cohort was projected to reach US$309.4 million by 2032. Psychiatric care would account for 19% of the all-cause care cost US$58.4 million. TRD states would account for 31.6% and 41.2% of total costs of all-cause and psychiatric care, and the new-onset comorbidities states would contribute to 54.0% and 46.1% respectively. In the first cycle, the annual cost of all-cause care would be US$39.5 million, which would drop to US$24.5 million by the tenth cycle at a compound annual growth rate (CAGR) of −5.1%. Similarly, the annual cost of psychiatric care would also drop from US$6.5 million to US$5.1 million at a CAGR of −2.6%. In the pandemic scenario in which 11,397 (18.8 per 10,000 population) newly diagnosed patients were modelled, the costs of both types of care would rise by 1.25-fold due to greater number of newly diagnosed patients (Fig. 3A).

#### Deaths, years of life lived and QALYs

After 10 cycles, 899 patients (case fatality rate: 9.8%) were projected to die. Causes of death of the reference cohorts are reported in Supplementary Table S12. Nearly half of the deaths (46.6%) would occur during the first three cycles (126–156 deaths per year) with a peak in the second cycle, whilst annual deaths would gradually decrease to 44 in the tenth cycle. With more new patients in the pandemic scenario, annual deaths in all cycles would rise by 25% (Supplementary Figure S2A). Attributed to all health states, TRD states or new-onset comorbidities states in the model, the total years of lives lived would be 85,495, 12,326 and 14,196 (pandemic scenario: 106,536, 15,359 and 17,690), and undiscounted QALYs would be 60,213, 6491 and 7748 (pandemic scenario: 75,032, 8088 and 9655) throughout the 10 cycles. Being diagnosed with depression, living with TRD and living with new-onset comorbidities therefore translate into reductions in quality of life by 29.6%, 47.3% and 45.4%, respectively.

### Subgroup analyses

**Fig. 4:**
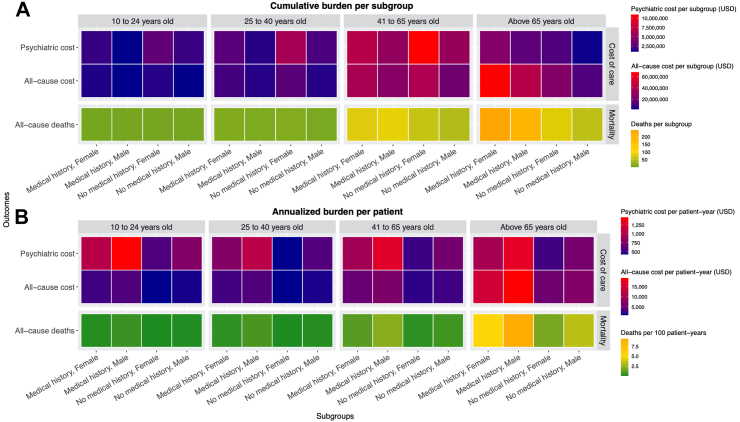
Projected costs and mortality burden by 2032 stratified by subgroups in the closed cohort model. The cumulative burden refers to the projected total collective costs of care or number of deaths accumulated in 10 years in one subgroup. The annualized burden per patient refers to the projected annual individual costs of care or the annual mortality rate, calculated by dividing the cumulative burden (nominator) by the years of lives lived (denominator) accumulated in 10 years in one subgroup. All costs are undiscounted and valued in 2023 U.S. Dollars (USD).

Cumulatively over the 10-year period, women diagnosed at ages above 65 with medical history collectively would incur the highest cost of all-cause care (US$68.0 million) and have the greatest number of deaths (n = 243). The highest cost of psychiatric care would be seen among women aged 41–65 without medical history at diagnosis, collectively reaching US$10.9 million (Fig. 4A). Regarding the individualised burden, men aged above 65 with medical history at diagnosis would have the highest cost of all-cause care (US$19,227 per patient-year) and highest number of deaths (55 deaths per 100 patient-years). The highest cost of psychiatric care would be seen among men aged between 10 and 24 with medical history at diagnosis (US$1452). Patients with medical history generally would have higher psychiatric cost regardless of age group and sex (Fig. 4B).

In addition, we observed that the proportion of real-world patients diagnosed at ages 10–24 increased to 17.0% among newly diagnosed patients in 2018, compared with 10.6% in the reference cohort diagnosed between 2014 and 2016. Applying the age and sex structure from 2018 increased the cumulative costs of all-cause and psychiatric care by 0.13% and 1.24% with differences of US$391,348 and US$725,197 respectively.

### Sensitivity analyses

**Fig. 5:**
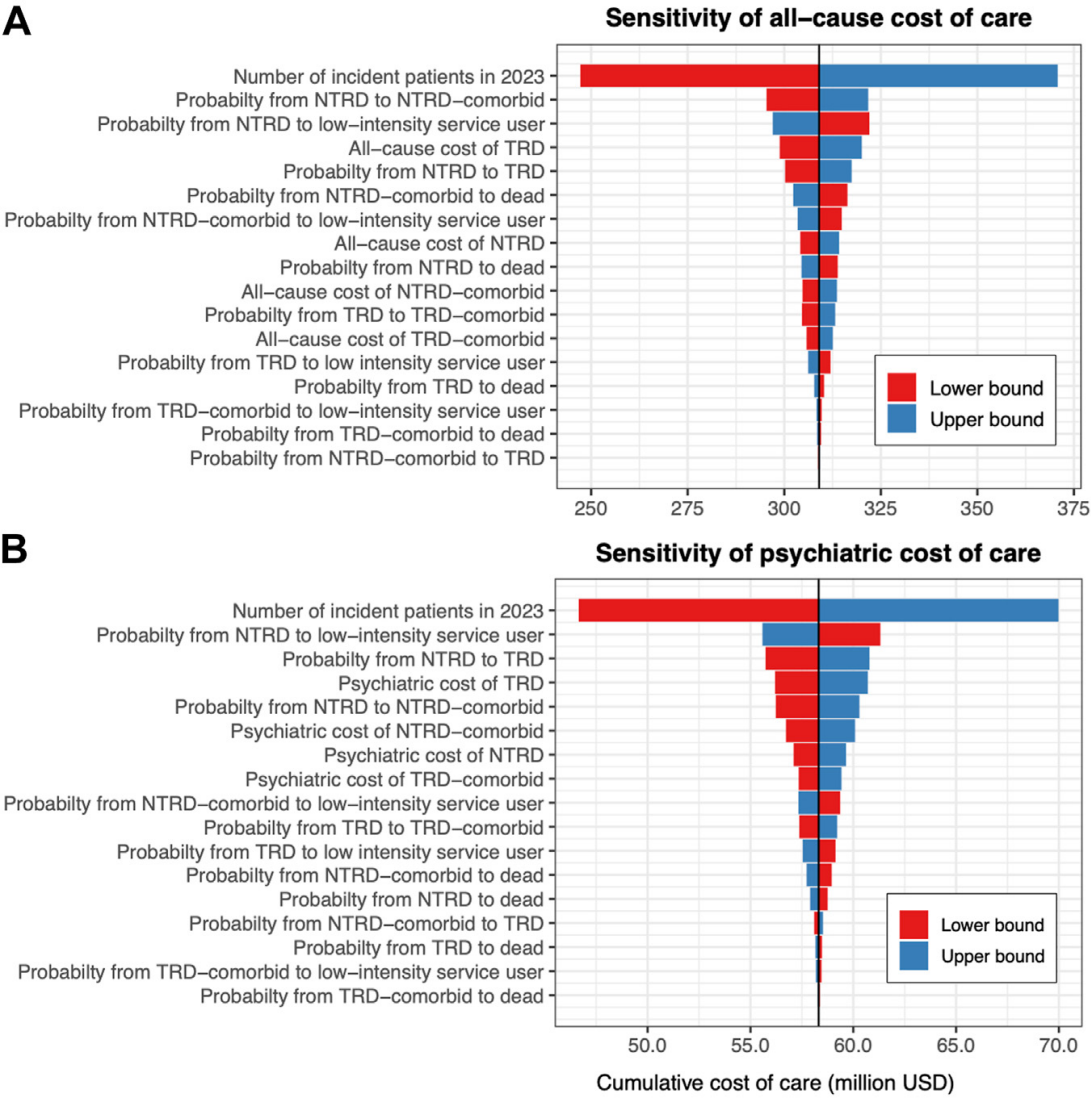
One-way deterministic sensitivity analyses for costs of all-cause and psychiatric care. Each bar represents the corresponding change in the costs of care when each parameter listed on the left side changes to its lower and upper bound. All costs are undiscounted and valued in 2023 U.S. Dollars (USD). Abbreviations: NTRD, Non-treatment-resistant depression; TRD, Treatment-resistant depression.

Our DSA showed that the number of incident patients had the most profound effect on the costs of both all-cause and psychiatric care, with the percentage reduction of newly diagnosed patients directly equivalent to the percentage of cost-savings. Other key drivers of cumulative costs included the probabilities of transiting from the NTRD state to onset of comorbidities, low-intensity service users and TRD states. Varying these parameters by 20% resulted in cost differences of between US$8.8 million and US$13.0 million (2.8–4.4% of base-case cost) for all-cause care and between US$2.1 million and US$3.0 million (3.6–5.2%) for psychiatric care (Fig. 5). In the PSA, the cumulative costs were US$311.2 million (SD ± US$33.0 million) and US$58.0 million (SD ± US$2.6 million) for all-cause and psychiatric care.

### Scenario analysis (open cohort model)

Between 2014 and 2032, the total number of patients who entered the model was 182,499 (pandemic scenario: 184,749) with 8397–11,705 new patients per year. In any current year between 2023 and 2032, we projected that 13.4–13.6% of prevalent cases would be living in the TRD states, and 15.4–15.6% would be living in new-onset comorbidities states. Among all prevalent cases diagnosed within 10 years, 6.4–6.7% new deaths would occur in any current year (Fig. 2B). Among 55,849–57,896 active patients living with depression in each year, the majority were women (73.4–73.5%) diagnosed between ages 41–65 (51.7–51.9%) without baseline medical history (69.8–70.0%).

Over the next 10 years, the annual undiscounted costs of all-cause care for prevalent cases were projected to be between US$322.3 million and US$336.8 million, whilst psychiatric care would continue to cost 19% of all-cause care (Fig. 3B). TRD states would account for 30.7–32.4% and 40.4–41.8% of all-cause and psychiatric care, and the new-onset comorbidities states would contribute to 53.7–54.4% and 45.7–46.5% of costs respectively. The pandemic scenario slightly increased the annual all-cause costs by 1.9–2.9% and psychiatric costs by 2.1–2.6%. In both scenarios, the 10-year costing trends were steady with CAGRs between −0.22% and −0.37%.

In any current year from 2023 to 2032, we projected 943–981 new deaths would occur from any causes across age groups, equivalent to 1.0% of the prevalent cases diagnosed within the previous 10 years. In the pandemic scenario, the number of annual deaths rose slightly by 1.1–3.9% to 954–1015 (Supplementary Figure S2B). Attributed to all states, TRD states or new-onset comorbidities states, the total years of lives lived were 913,762, 132,106 and 151,718 (pandemic scenario: 934,799, 135,139 and 155,211), and undiscounted QALYs were 643,657, 69,566 and 82,787 (pandemic scenario: 658,474, 71,163 and 84,703) over the 10-year period, implying reductions in quality of life within magnitudes similar to those reported in the closed cohort setting.

### Discounted outcomes

Simulated outcomes with discounting applied are listed in Supplementary Tables S13A–B.

## Discussion

In contrast to trial-based modelling, this study incorporated real-world data to address the roles of TRD and comorbidities on care burden and enhance generalisability of modelled outcomes to the local economic and clinical context. With 9217 new patients in 2023, our closed cohort model projected the 10-year undiscounted cumulative costs of all-cause and psychiatric care to reach US$309 million and US$58 million, respectively, with 899 cumulative deaths by 2032. The open cohort model showed that 55,849–57,896 active prevalent cases would cost more than US$322 million and US$61 million in all-cause and psychiatric care with 943–981 deaths annually. TRD and comorbidity development would contribute to substantially to costs. Middle-aged and older women collectively would incur the highest cumulative burden, whilst older and younger men with medical history would have the greatest individualised burden.

Our estimates were consistent with the historical real-world local context. In the open cohort model, we projected 56,000–58,000 active prevalent cases remaining in the system each year, which accounted for 21% of all patients with mental illnesses using public psychiatric services in 2020/2021.^45^ This figure broadly aligns with the number of patients with depression (64,700) in 2021/2022 reported by government, with the numeric difference attributable to our exclusion of patients aged under 10 and diagnosed more than 10 years previously.^46^ Locally, a cross-sectional survey studying community-dwelling older adults estimated that the annual cost of all-cause healthcare and rehabilitation was US$6230 (valued in 2017/2018) among patients with mild depression.^47^ Despite non-comparable results due to different study designs and patient categories, our study projected consistent costs of all-cause care per patient-year among older patients without medical history (US$5349 and US$7030 in women and men). Our findings support the reliability of previous local analyses. We also examined the roles of developing TRD and comorbidities during the disease course. Although the proportions of patients living with TRD or comorbidities were below 20% in all cycles, these two states individually contributed to 31–54% of the costs. This concurs with previous studies in the U.S., Japan, South Korea, and Hong Kong, which found that TRD was associated with 1.4- to 4-fold higher medical costs compared with treatment-responsive depression.^13^^,^^48^, ^49^, ^50^, ^51^, ^52^ Another study in the U.S. also showed a greater proportion of incremental cost due to comorbidities than to depression alone.^11^ These patients were not the majority, but our results add to highlight the significant costs of these conditions due to heightened care need, which should be considered when developing cost-saving strategies and planning forthcoming care needs.

We projected gradually declining costs and all-cause deaths over the next decade. In the closed cohort model, the trend was legitimate by aligning with the disease course, during which patients from a fixed cohort eventually died, became low-intensity service users, or remained as stable depression cases. The highest cost occurred in the first year of diagnosis, whilst the greatest deaths occurred in the second year as it takes time for disease progression, in contrast to an immediate effect in costs that was driven by frequency of healthcare service use. After 10 cycles, however, costs only decreased by 38% and 21% in all-cause and psychiatric care, which suggests a sustained care burden and disease chronicity. In the open cohort model, in contrast, projected trends of annual costs were steady despite an increase in the number of new patients driven by population growth over time. This could be caused by the spike of depression cases between 2019 and 2022, the burden from which was sustained in the first years of projection before stabilising and exiting the model. This also echoes with our sensitivity analysis, in which the number of new patients had the most pronounced effect for costs. Other key cost drivers included the transitions from the NTRD state to TRD, comorbidities and low-intensity service user states. A common feature of these factors was their relevance to the early stages of disease, which implies early treatments and interventions that reduce the risk of depression could lead to cost-savings, particularly given the immense burden once patients acquired TRD or comorbidities. Examples of intervention include community peers support and cognitive behavioural therapy-based prevention that targets universal population and subthreshold depression, and early accessible community and medical assistance in newly diagnosed depression.^53^ Multidisciplinary care is also crucial to detect comorbidities early. Although not reported here, our projection model could be applied to estimate cost-savings by setting hypothetical scenarios of reduced number of patients specific to TRD and/or comorbidities for resource planning.

Our subgroup analysis helped identify patient characteristics associated with the highest level of resource use. In terms of cumulative burden, middle-aged and older women collectively had the highest costs since the distribution was likely to reflect a greater proportion of middle-aged and older women in the actual patient population. After annualising and individualising the burden, older men with medical history had the highest cost of all-cause care and most deaths. It is well-established that old age and medical history is associated with greater care burden.^54^ Compared with women who were also in the oldest age group with medical history, our estimated transition probabilities show that men appeared to have greater chances to transit into comorbidity states and also more likely to die after comorbidities. This also aligns with prior findings of a higher risk of depression in women, but generally higher risks of self-harm and development of physical comorbidities in men once they were diagnosed with depression.^19^^,^^55^ Surprisingly, children/adolescent men with medical history had the highest cost of psychiatric care. One possible reason could be a longer duration of depression episode in children than adults.^56^ A previous meta-analysis also revealed that adolescent depression led to highest excess costs than other age groups.^6^ Although it is unclear if the young were more resource-intensive in receiving treatments or the older patients under-utilised psychiatric care, an unavoidable implication is to increase attention to these two age groups with foci on reduction in overall risk of depression and comorbidities management.

Our study has both strengths and limitations. Since our projected outcomes reflect future costs, years of lives lived and QALYs under the status quo, we have created a basis for future analyses of cost-effectiveness of innovative medicines compared with the current depression treatments. Incorporating local real-world data when deriving most model inputs, our findings also inform future budget planning in depression care, which is highly generalisable to the actual context, and support the identification of potential unmet needs for intervention. Moreover, this is the first model to adapt TRD and comorbidities as health states and study their intensifying effect in cost and mortality burden with the help of real-world data. We acknowledge limitations of our study. Firstly, as with other EMR-based studies, we lacked psychometric details and clinical notes to understand symptom severity, as well as rationales of treatment choices and prescribed dosages, making it difficult to distinguish if the changes in medications were due to undertreatment, pharmacokinetics, adverse events, or lack of treatment responses. We cannot completely rule out pseudo-resistance. Without a consensus on TRD definition, we followed the most widely used definitions stated by the U.S. Food and Drug Administration and European Medicines Agency.^57^ The role of psychotherapies is however not considered in these definitions, which possibly leads to a conservative estimate of patients resistant to overall types of treatment. Secondly, though local clinicians expressed interests in modelling suicides, it was technically challenging due to incomplete coding of the cause of death data. Third, we derived cost and probability inputs from the follow-up of the 2014–2016 cohorts, which could lead to different findings if the future real-world cohorts have different patterns of healthcare service use, disease course or treatment strategies. Lastly, out study was limited to modelling the direct burden of diagnosed depression and did not account for utilization and higher costs in patients who self-treated or sought medical care for somatic problems without ever being diagnosed with depression. As we set out from a payer perspective instead of a societal perspective, the substantial indirect burden such as productivity losses were not studied and the presented total cost was likely an underestimate.

## Conclusion

Depression presents significant risks of mortality, healthcare costs and quality of life burdens to society and healthcare system. Our projected estimates of these impacts over a 10-year period provide insights to support resource allocation and budget planning to prepare for future care needs under alternative scenarios. The use of real-world data revealed that TRD and comorbidity development contributed to substantial costs in the real-world setting, which calls for appropriate resource allocation for TRD and multi-disciplinary care for depression-related comorbidities.

## Contributors

X Li conceived the study idea and study design. VKY Chan and D Yang gathered the data and performed data analyses. M Leung, SSM Chan, M Knapp and H Luo provided technical advice from health economics, clinical psychiatry, and statistical disciplines. All authors participated in protocol development and/or interpreted the results. VKY Chan and X Li wrote and revised the drafts with all authors’ critical comments and revisions. All authors approved the final version for manuscript submission. All authors agree to be accountable for all aspects of the work. X Li and ICK Wong obtained the funding and supervised the study conduct. The corresponding authors confirm that all co-authors meet authorship criteria.

## Data sharing statement

We are unable to directly share the data used in this study since the data custodian, the Hong Kong Hospital Authority who manages the Clinical Data Analysis and Reporting System (CDARS), has not given permission. However, CDARS data can be accessed and requested via the Hospital Authority Data Sharing Portal for research purpose. The relevant information can be found online (https://www3.ha.org.hk/data). The analysis plan, model, statistical procedures and programming codes used in this study are available on GitHub and Dataverse via https://github.com/scan2030 and https://doi.org/10.7910/DVN/ZVBFVA.

## Editor note

The Lancet Group takes a neutral position with respect to territorial claims in published maps and institutional affiliations.

## Declaration of interests

X Li received research grants from Hong Kong Health and Medical Research Fund (HMRF, HMRF Fellowship Scheme, HKSAR), Research Grants Council Early Career Scheme (RGC/ECS, HKSAR), Janssen and Pfizer; internal funding from the University of Hong Kong; consultancy fees from Merck Sharp & Dohme and Pfizer; she is also a non-executive director of Advanced Data Analytics for Medical Science (ADAMS) Limited Hong Kong, all are unrelated to this work; H Luo received research grants Research Grants Council Early Career Scheme (RGC/ECS, HKSAR) unrelated to this work. EWY Chan reports grants from Research Grants Council, Research Fund Secretariat of the Food and Health Bureau, National Natural Science Fund of China, Wellcome Trust, Bayer, Amgen, Bristol-Myers Squibb, Janssen, Takeda, Narcotics Division of the Security Bureau of Hong Kong, honorarium from Hospital Authority, outside the submitted work; ICK Wong received research funding outside the submitted work from Amgen, Bristol-Myers Squibb, Pfizer, Janssen, Bayer, GSK, Novartis, Takeda, the Hong Kong RGC, and the Hong Kong Health and Medical Research Fund, National Institute for Health Research in England, European Commission, National Health and Medical Research Council in Australia, The European Union's Seventh Framework Programme for research technological development, and has also received consulting fees from IQVIA, the WHO and expert testimony for Appeal Court in Hong Kong over the past three years. He is also a non-executive director of Jacobson Medical Hong Kong, and founder and director of Therakind Limited (United Kingdom), Advanced Data Analytics for Medical Science (ADAMS) Limited (Hong Kong), Asia Medicine Regulatory Affairs (AMERA) Services Limited and OCUS Innovation Limited (Hong Kong, Ireland and United Kingdom).
