# Supplementary material for: Projecting the 10-year costs of care and mortality burden of depression until 2032: a Markov modelling study developed from real-world data
Source: Lancet Reg Health West Pac. 2024 Feb 6;45:101026. doi: 10.1016/j.lanwpc.2024.101026 (PMC10862399; doi:10.1016/j.lanwpc.2024.101026)
Supplement: Supplementary Tables and Figures [file mmc1.docx]

**Supplementary Materials Legend**

**Tables**

Supplementary Table 1 – Detailed definitions of health states in the Markov model

Supplementary Table 2A – Model input parameters, costs of care

Supplementary Table 2B – Model input parameters, utilities, number of new patients and subgroup weights

Supplementary Table 2C – Model input parameters, transition probabilities

Supplementary Table 3 – Medical conditions to define baseline medical history and new-onset comorbidities

Supplementary Table 4 – Akaike and Bayesian Information Criterion values

Supplementary Table 5 – Modelled and observed cumulative numbers of deaths and low-intensity service users for validation of derived transition probabilities

Supplementary Table 6 – Mean absolute percentage errors of predicted deaths at cycle 4 in parametric survival modelling

Supplementary Table 7 – Mean absolute percentage errors of predicted low-intensity service users at cycle 4 in parametric survival modelling

Supplementary Table 8 – Non-subsidised unit costs of service types in the public healthcare system of Hong Kong, adapted from Hospital Authority website in 2023

Supplementary Table 9 – Justification of adapted utility weights

Supplementary Table 10A – Quality assessment for the selected HRQoL articles, cross-sectional studies

Supplementary Table 10B – Quality assessment for the selected HRQoL articles, cohort study

Supplementary Table 10C – Quality assessment for the selected HRQoL articles, randomized controlled trial

Supplementary Table 11 – Baseline characteristics of patients newly diagnosed with incident depression between 2014 and 2016 at cohort entry

Supplementary Table 12 – Causes of death in the 2014-2016 incident cohorts

Supplementary Table 13A – Undiscounted and discounted projected outcomes, base-case scenario (closed cohort model)

Supplementary Table 13B – Undiscounted and discounted projected outcomes, scenario analysis (open cohort model)

**Figures**

Supplementary Figure 1 – Schematic presentation of scope of included patients in the closed and open cohort models

Supplementary Figure 2 – Projected annual numbers of all-cause death from 2023 to 2032

**Texts**

Supplementary Methods – Detailed description on derivation of model input parameters

**Supplementary Table 1.** Detailed definitions of health states in the Markov model

| **Health states** | **Definitions** |
| --- | --- |
| Non-treatment-resistant depression (NTRD) | Patients with depression who were yet to develop TRD or further clinical characteristics. This was also the initial health state where patients with newly diagnosed depression stayed before transiting to other health states. |
| Treatment-resistant depression (TRD) | Patients who took at least two antidepressant regimens for an adequate duration and had the third regimen to confirm refractoriness in the first two regimens throughout the follow-up. An adequate duration refers to the same antidepressant or combination regimen of at least 28 days with gaps no longer than 14 days within regimens. |
| New-onset comorbidities (NTRD-comorbid) | Patients with new-onset somatic comorbidities included in the list of diseases used to calculate Charlson Comorbidity Index, or pre-specified psychiatric comorbidities before TRD, and the new-onset condition(s) did not occur before depression diagnosis. The list of included somatic and psychiatric conditions are shown in Supplementary Table 3. |
| New-onset post-TRD comorbidities (TRD-comorbid) | Patients with new-onset somatic comorbidities included in the list of diseases used to calculate Charlson Comorbidity Index, or pre-specified psychiatric comorbidities only after developing TRD, and the new-onset condition(s) did not occur before TRD development. The list of somatic and psychiatric conditions is shown in Supplementary Table 3. |
| Low-intensity service user (absorbing state) | Patients with minimal care need and free of further depression-related diagnosis records and antidepressant prescriptions. This was defined as having at least 1-year absence of depression-related diagnosis records across the outpatient, inpatient and emergency settings and no prescription records of antidepressants, and the absence of records continued until the end of follow-up. The date of becoming low-intensity user was estimated as the last date of prescription or 180 days after the last depression-related diagnosis. The health state acts as a proxy for recovery from depression. Patients in this state are not considered as active or living with depression, therefore costs and mortality that occurred during this state were not counted towards depression burden in the healthcare system. |
| All-cause death (absorbing state) | Observable deaths regardless of causes. |

**Supplementary Table 2A.** Model input parameters, cost of care

| **Health state** | **Age group*** | **Sex** | **Baseline medical history*** | **All-cause value** | **Lower bound** | **Upper bound** | **Psychiatric value** | **Lower bound** | **Upper bound** |
| --- | --- | --- | --- | --- | --- | --- | --- | --- | --- |
| NTRD | 10-24 | Female | No | 7491 | 6988 | 8031 | 4220 | 3815 | 4668 |
|  | 10-24 | Female | Yes | 19954 | 18422 | 21614 | 7630 | 6795 | 8568 |
|  | 10-24 | Male | No | 9644 | 8914 | 10433 | 5738 | 5119 | 6432 |
|  | 10-24 | Male | Yes | 25689 | 23566 | 28002 | 10376 | 9156 | 11759 |
|  | 25-40 | Female | No | 8050 | 7682 | 8436 | 3167 | 2959 | 3390 |
|  | 25-40 | Female | Yes | 21445 | 20153 | 22819 | 5727 | 5233 | 6267 |
|  | 25-40 | Male | No | 10364 | 9738 | 11030 | 4307 | 3935 | 4714 |
|  | 25-40 | Male | Yes | 27607 | 25676 | 29683 | 7788 | 7010 | 8652 |
|  | 41-65 | Female | No | 12200 | 11719 | 12701 | 3075 | 2901 | 3260 |
|  | 41-65 | Female | Yes | 32498 | 30757 | 34338 | 5561 | 5134 | 6023 |
|  | 41-65 | Male | No | 15706 | 14864 | 16596 | 4182 | 3860 | 4530 |
|  | 41-65 | Male | Yes | 41837 | 39226 | 44622 | 7562 | 6887 | 8303 |
|  | 65+ | Female | No | 30660 | 28580 | 32891 | 2746 | 2480 | 3041 |
|  | 65+ | Female | Yes | 81671 | 76154 | 87587 | 4965 | 4486 | 5496 |
|  | 65+ | Male | No | 39470 | 36418 | 42779 | 3734 | 3322 | 4197 |
|  | 65+ | Male | Yes | 105140 | 97216 | 113710 | 6752 | 6026 | 7565 |
| NTRD-comorbid | 10-24 | Female | No | 20784 | 18965 | 22776 | 11284 | 9880 | 12887 |
|  | 10-24 | Female | Yes | 55363 | 50154 | 61113 | 20403 | 17678 | 23549 |
|  | 10-24 | Male | No | 26756 | 24290 | 29473 | 15344 | 13335 | 17656 |
|  | 10-24 | Male | Yes | 71272 | 64351 | 78939 | 27746 | 23923 | 32180 |
|  | 25-40 | Female | No | 22336 | 20676 | 24130 | 8469 | 7571 | 9473 |
|  | 25-40 | Female | Yes | 59498 | 54570 | 64870 | 15314 | 13509 | 17361 |
|  | 25-40 | Male | No | 28755 | 26403 | 31315 | 11517 | 10176 | 13034 |
|  | 25-40 | Male | Yes | 76595 | 69854 | 83988 | 20825 | 18219 | 23804 |
|  | 41-65 | Female | No | 33849 | 31592 | 36267 | 8223 | 7439 | 9089 |
|  | 41-65 | Female | Yes | 90166 | 83441 | 97432 | 14869 | 13287 | 16639 |
|  | 41-65 | Male | No | 43576 | 40381 | 47024 | 11182 | 10012 | 12488 |
|  | 41-65 | Male | Yes | 116077 | 106922 | 126015 | 20220 | 17948 | 22780 |
|  | 65+ | Female | No | 85065 | 78278 | 92441 | 7343 | 6508 | 8284 |
|  | 65+ | Female | Yes | 226594 | 208775 | 245932 | 13278 | 11790 | 14953 |
|  | 65+ | Male | No | 109510 | 100097 | 119809 | 9985 | 8764 | 11376 |
|  | 65+ | Male | Yes | 291709 | 267393 | 318237 | 18056 | 15913 | 20486 |
| TRD | 10-24 | Female | No | 15943 | 14689 | 17304 | 12100 | 10744 | 13628 |
|  | 10-24 | Female | Yes | 42468 | 38792 | 46492 | 21880 | 19187 | 24952 |
|  | 10-24 | Male | No | 20524 | 18762 | 22452 | 16455 | 14445 | 18744 |
|  | 10-24 | Male | Yes | 54671 | 49659 | 60190 | 29754 | 25879 | 34210 |
|  | 25-40 | Female | No | 17133 | 16082 | 18253 | 9082 | 8285 | 9956 |
|  | 25-40 | Female | Yes | 45639 | 42344 | 49191 | 16423 | 14730 | 18309 |
|  | 25-40 | Male | No | 22057 | 20453 | 23787 | 12350 | 11069 | 13780 |
|  | 25-40 | Male | Yes | 58755 | 54035 | 63886 | 22333 | 19777 | 25218 |
|  | 41-65 | Female | No | 25965 | 24472 | 27548 | 8818 | 8092 | 9609 |
|  | 41-65 | Female | Yes | 69164 | 64510 | 74154 | 15945 | 14412 | 17641 |
|  | 41-65 | Male | No | 33426 | 31162 | 35856 | 11991 | 10831 | 13276 |
|  | 41-65 | Male | Yes | 89040 | 82417 | 96195 | 21684 | 19383 | 24257 |
|  | 65+ | Female | No | 65251 | 60097 | 70848 | 7874 | 6988 | 8873 |
|  | 65+ | Female | Yes | 173815 | 160187 | 188601 | 14239 | 12648 | 16030 |
|  | 65+ | Male | No | 84003 | 76682 | 92022 | 10708 | 9381 | 12223 |
|  | 65+ | Male | Yes | 223764 | 204718 | 244581 | 19363 | 17018 | 22030 |
| TRD-comorbid | 10-24 | Female | No | 44233 | 40063 | 48836 | 32357 | 28027 | 37356 |
|  | 10-24 | Female | Yes | 117826 | 106032 | 130931 | 58509 | 50207 | 68185 |
|  | 10-24 | Male | No | 56944 | 51330 | 63172 | 44001 | 37849 | 51152 |
|  | 10-24 | Male | Yes | 151685 | 136074 | 169087 | 79565 | 67964 | 93145 |
|  | 25-40 | Female | No | 47536 | 43636 | 51785 | 24286 | 21449 | 27498 |
|  | 25-40 | Female | Yes | 126625 | 115302 | 139061 | 43915 | 38334 | 50309 |
|  | 25-40 | Male | No | 61197 | 55764 | 67158 | 33025 | 28858 | 37794 |
|  | 25-40 | Male | Yes | 163014 | 147656 | 179969 | 59718 | 51731 | 68938 |
|  | 41-65 | Female | No | 72039 | 66588 | 77936 | 23580 | 21036 | 26432 |
|  | 41-65 | Female | Yes | 191895 | 176103 | 209103 | 42639 | 37643 | 48297 |
|  | 41-65 | Male | No | 92741 | 85182 | 100970 | 32066 | 28344 | 36276 |
|  | 41-65 | Male | Yes | 247040 | 225757 | 270329 | 57983 | 50877 | 66080 |
|  | 65+ | Female | No | 181039 | 165278 | 198303 | 21056 | 18449 | 24031 |
|  | 65+ | Female | Yes | 482246 | 440916 | 527450 | 38075 | 33433 | 43361 |
|  | 65+ | Male | No | 233064 | 211463 | 256872 | 28633 | 24865 | 32974 |
|  | 65+ | Male | Yes | 620828 | 564949 | 682234 | 51776 | 45154 | 59370 |

*At diagnosis. Costs in Hong Kong Dollars valued in 2023. Values are estimated from cost analysis of the real-world 2014-2016 incident cohorts with details described in the main article and Supplementary Methods. Lower and upper bounds were based on the 95% confidence interval of regression coefficients. Gamma distribution was used in the probabilistic sensitivity analysis. Abbreviations: NTRD – Non-treatment-resistant depression, TRD – treatment-resistant depression.

**Supplementary Table 2B.** Model input parameters, utilities, number of new patients and subgroup weights

| **Inputs** | **Value** | **Range or variation** | **Distribution** | **Rationales and remarks** |
| --- | --- | --- | --- | --- |
| **Utility per health state** | | | | |
| NTRD | 0.68 | 0.60 - 0.72 | Beta | Reference 1 |
| TRD | 0.54 | 0.42 - 0.60 | Beta | Reference 1 |
| NTRD-comorbid | 0.56 | 0.50 - 0.60 | Beta | Reference 2 to 5 |
| TRD-comorbid | 0.45 | 0.35 - 0.48 | Beta | Reference 2 to 5 |
| Low-intensity service user | 0.85 | 0.52 - 0.88 | Beta | Reference 6 |
| **Number of incident patients** | | | | |
| Year 2014 | 8,657 | NA | Actual value | Actual number of newly diagnosed patients in the corresponding year. |
| Year 2015 | 8,728 | NA | Actual value |  |
| Year 2016 | 8,397 | NA | Actual value |  |
| Year 2017 | 8,630 | NA | Actual value |  |
| Year 2018 | 8,463 | NA | Actual value |  |
| Year 2019^a^ | 11,705 | NA | Not assigned^c^ | Projected based on the actual age-specific mid-year population and the age-specific annual incidences recorded between 2014 and 2018, accounting for increase due to the social movement in 2019 and pandemic between 2020 and 2022/2023^7^. |
| Year 2020^a^ | 10,964 | NA | Not assigned^c^ |  |
| Year 2021^a^ | 10,988 | NA | Not assigned^c^ |  |
| Year 2022^a^ | 10,885 | NA | Not assigned^c^ |  |
| Year 2023^b^ | 9,147 / 11,398 | -20% to +20% | Poisson |  |
| Year 2024 | 9,238 | NA | Not assigned^c^ | Projected based on the age-specific mid-year population projections released by government and the age-specific annual incidences recorded between 2014 and 2018, assuming no residual impact due to pandemic and social movement. |
| Year 2025 | 9,327 | NA | Not assigned^c^ |  |
| Year 2026 | 9,414 | NA | Not assigned^c^ |  |
| Year 2027 | 9,496 | NA | Not assigned^c^ |  |
| Year 2028 | 9,559 | NA | Not assigned^c^ |  |
| Year 2029 | 9,625 | NA | Not assigned^c^ |  |
| Year 2030 | 9,696 | NA | Not assigned^c^ |  |
| Year 2031 | 9,758 | NA | Not assigned^c^ |  |
| Year 2032 | 9,822 | NA | Not assigned^c^ |  |
| **Subgroup weights** | | | | |
| 10-24 years old, Women, No medical history | 0.056649 | NA | Dirichlet | Actual subgroup distributions observed in the real-world 2014-2016 incident cohorts. |
| 25-40 years old, Women, No medical history | 0.153593 | NA | Dirichlet |  |
| 41-65 years old, Women, No medical history | 0.238547 | NA | Dirichlet |  |
| 65+ years old, Women, No medical history | 0.054148 | NA | Dirichlet |  |
| 10-24 years old, Women, Medical history | 0.017825 | NA | Dirichlet |  |
| 25-40 years old, Women, Medical history | 0.037912 | NA | Dirichlet |  |
| 41-65 years old, Women, Medical history | 0.085987 | NA | Dirichlet |  |
| 65+ years old, Women, Medical history | 0.069194 | NA | Dirichlet |  |
| 10-24 years old, Men, No medical history | 0.024772 | NA | Dirichlet |  |
| 25-40 years old, Men, No medical history | 0.041723 | NA | Dirichlet |  |
| 41-65 years old, Men, No medical history | 0.086145 | NA | Dirichlet |  |
| 65+ years old, Men, No medical history | 0.021159 | NA | Dirichlet |  |
| 10-24 years old, Men, Medical history | 0.007900 | NA | Dirichlet |  |
| 25-40 years old, Men, Medical history | 0.015284 | NA | Dirichlet |  |
| 41-65 years old, Men, Medical history | 0.048511 | NA | Dirichlet |  |
| 65+ years old, Men, Medical history | 0.040651 | NA | Dirichlet |  |

^a.^ A major social movement occurred in Hong Kong between June and December 2019, followed by the COVID-19 pandemic between January 2020 and December 2022. We therefore added the excess new cases based on the reported increase in the prevalence of probable depression from a territory-wide large-scale local prospective cohort study. We assumed 1.72- and 1.53-fold increase in new cases between 2019 and 2022, with 52.5% of excess cases who eventually sought medical help and entered the public healthcare system.

^b.^ Two sets of number of newly diagnosed patients were estimated for 2023 to address potential residual pandemic impact.

^c.^ No distribution was assigned since the inputs were not involved in the probabilistic sensitivity analysis of the base-case scenario.

NA: Input parameters were either not involved in the base-case scenario, or one-way deterministic sensitivity analysis was not possible to only vary one parameter without changing others.

Abbreviations: NA – Not applicable, NTRD – Non-treatment-resistant depression, TRD – treatment-resistant depression.

References:

1. Rathod S, Denee T, Eva J, et al. Health-related quality of life burden associated with treatment-resistant depression in UK patients: Quantitative results from a mixed-methods non-interventional study. *J Affect Disord*. 2022;300:551-562.
2. Johansson R, Carlbring P, Heedman Å, Paxling B, Andersson G. Depression, anxiety and their comorbidity in the Swedish general population: point prevalence and the effect on health-related quality of life. *PeerJ*. 2013;1:e98.
3. Zhou Y, Cao Z, Yang M, et al. Comorbid generalized anxiety disorder and its association with quality of life in patients with major depressive disorder. *Sci Rep*. 2017;7:40511.
4. IsHak WW, Steiner AJ, Klimowicz A, et al. Major Depression Comorbid with Medical Conditions: Analysis of Quality of Life, Functioning, and Depressive Symptom Severity. *Psychopharmacol Bull*. 2018;48(1):8-25.
5. Moussavi S, Chatterji S, Verdes E, Tandon A, Patel V, Ustun B. Depression, chronic diseases, and decrements in health: results from the World Health Surveys. *Lancet*. 2007;370(9590):851-858.
6. Sapin C, Fantino B, Nowicki ML, Kind P. Usefulness of EQ-5D in assessing health status in primary care patients with major depressive disorder. *Health Qual Life Outcomes*. 2004;2:20.
7. Ni MY, Yao XI, Leung KSM, et al. Depression and post-traumatic stress during major social unrest in Hong Kong: a 10-year prospective cohort study. *Lancet*. 2020;395(10220):273-284.

**Supplementary Table 2C**. Model input parameters, transition probabilities

| **Cycle** | **tpNTRD**  **2TRD** | **tpNTRD**  **2COM** | **tpNTRD**  **2DEAD** | **tpNTRD**  **2LOW** | **tpTRD**  **2TCOM** | **tpTRD**  **2DEAD** | **tpTRD**  **2LOW** | **tpCOM**  **2TRD** | **tpCOM**  **2DEAD** | **tpCOM**  **2LOW** | **tpTCOM**  **2DEAD** | **tpTCOM**  **2LOW** |
| --- | --- | --- | --- | --- | --- | --- | --- | --- | --- | --- | --- | --- |
| 10-24 years old, Women, No medical history* | | | | | | | | | | | | |
| 1 | 6.6697% | 17.7479% | 0.0358% | 19.5508% | 9.5837% | 0.128% | 7.9010% | 10.9845% | 0.3120% | 16.7702% | 0.5587% | 11.0122% |
| 2 | 6.4386% | 5.5400% | 0.0358% | 17.6739% | 6.8047% | 0.128% | 9.2747% | 7.3374% | 0.3111% | 14.3274% | 0.1881% | 7.6989% |
| 3 | 5.6091% | 3.9944% | 0.0358% | 14.7929% | 6.0379% | 0.127% | 9.8728% | 5.7908% | 0.3101% | 11.8635% | 0.1381% | 6.1373% |
| 4 | 4.9957% | 3.2322% | 0.0358% | 12.8307% | 5.5869% | 0.127% | 10.2813% | 4.8863% | 0.3091% | 10.2416% | 0.1132% | 5.2061% |
| 5 | 4.5287% | 2.7612% | 0.0358% | 11.4049% | 5.2733% | 0.127% | 10.5955% | 4.2740% | 0.3082% | 9.0800% | 0.0977% | 4.5693% |
| 6 | 4.1595% | 2.4353% | 0.0358% | 10.3137% | 5.0358% | 0.127% | 10.8523% | 3.8247% | 0.3072% | 8.1983% | 0.0869% | 4.0989% |
| 7 | 3.8586% | 2.1936% | 0.0358% | 9.4462% | 4.8462% | 0.127% | 11.0704% | 3.4774% | 0.3063% | 7.5010% | 0.0788% | 3.7336% |
| 8 | 3.6074% | 2.0057% | 0.0358% | 8.7366% | 4.6895% | 0.127% | 11.2604% | 3.1989% | 0.3054% | 6.9326% | 0.0725% | 3.4397% |
| 9 | 3.3939% | 1.8545% | 0.0357% | 8.1430% | 4.5566% | 0.126% | 11.4290% | 2.9696% | 0.3044% | 6.4585% | 0.0674% | 3.1968% |
| 10 | 3.2095% | 1.7298% | 0.0357% | 7.6378% | 4.4416% | 0.126% | 11.5808% | 2.7766% | 0.3035% | 6.0558% | 0.0632% | 2.9921% |
| 25-40 years old, Women, No medical history* | | | | | | | | | | | | |
| 1 | 7.2949% | 10.9742% | 0.0690% | 15.1151% | 4.5040% | 0.241% | 4.9008% | 8.9812% | 0.3945% | 12.6488% | 0.7681% | 7.3431% |
| 2 | 6.8825% | 3.3341% | 0.0689% | 14.7973% | 3.1724% | 0.240% | 5.7693% | 6.3047% | 0.3929% | 11.7341% | 0.2587% | 5.6755% |
| 3 | 5.9476% | 2.3961% | 0.0689% | 12.6917% | 2.8087% | 0.240% | 6.1491% | 5.0503% | 0.3914% | 9.9696% | 0.1900% | 4.6678% |
| 4 | 5.2725% | 1.9358% | 0.0688% | 11.1577% | 2.5956% | 0.239% | 6.4090% | 4.2993% | 0.3899% | 8.7322% | 0.1558% | 4.0334% |
| 5 | 4.7642% | 1.6521% | 0.0688% | 10.0077% | 2.4477% | 0.239% | 6.6092% | 3.7839% | 0.3884% | 7.8181% | 0.1344% | 3.5859% |
| 6 | 4.3652% | 1.4561% | 0.0687% | 9.1105% | 2.3359% | 0.238% | 6.7732% | 3.4020% | 0.3869% | 7.1105% | 0.1195% | 3.2483% |
| 7 | 4.0417% | 1.3109% | 0.0687% | 8.3875% | 2.2468% | 0.238% | 6.9125% | 3.1048% | 0.3854% | 6.5430% | 0.1084% | 2.9820% |
| 8 | 3.7727% | 1.1982% | 0.0686% | 7.7901% | 2.1732% | 0.237% | 7.0339% | 2.8651% | 0.3839% | 6.0754% | 0.0997% | 2.7650% |
| 9 | 3.5447% | 1.1075% | 0.0686% | 7.2864% | 2.1108% | 0.236% | 7.1418% | 2.6667% | 0.3824% | 5.6821% | 0.0927% | 2.5839% |
| 10 | 3.3483% | 1.0328% | 0.0685% | 6.8548% | 2.0569% | 0.236% | 7.2390% | 2.4991% | 0.3810% | 5.3455% | 0.0869% | 2.4299% |
| 41-65 years old, Women, No medical history* | | | | | | | | | | | | |
| 1 | 5.4763% | 12.4641% | 0.1963% | 7.3968% | 7.1573% | 0.298% | 3.1307% | 6.7684% | 1.3440% | 5.6741% | 2.7714% | 5.4549% |
| 2 | 5.5549% | 3.8088% | 0.1959% | 8.9408% | 5.0622% | 0.297% | 3.6917% | 5.0850% | 1.3262% | 6.5631% | 0.9399% | 4.5240% |
| 3 | 4.9264% | 2.7392% | 0.1956% | 8.2479% | 4.4871% | 0.296% | 3.9375% | 4.1608% | 1.3088% | 6.0265% | 0.6910% | 3.8087% |
| 4 | 4.4337% | 2.2137% | 0.1952% | 7.5508% | 4.1493% | 0.295% | 4.1060% | 3.5872% | 1.2919% | 5.5172% | 0.5667% | 3.3371% |
| 5 | 4.0482% | 1.8897% | 0.1948% | 6.9584% | 3.9147% | 0.294% | 4.2359% | 3.1853% | 1.2754% | 5.0900% | 0.4891% | 2.9959% |
| 6 | 3.7383% | 1.6658% | 0.1944% | 6.4617% | 3.7372% | 0.294% | 4.3424% | 2.8832% | 1.2594% | 4.7333% | 0.4350% | 2.7340% |
| 7 | 3.4826% | 1.4998% | 0.1940% | 6.0417% | 3.5956% | 0.293% | 4.4329% | 2.6456% | 1.2437% | 4.4322% | 0.3947% | 2.5247% |
| 8 | 3.2674% | 1.3710% | 0.1937% | 5.6821% | 3.4786% | 0.292% | 4.5118% | 2.4523% | 1.2284% | 4.1743% | 0.3631% | 2.3525% |
| 9 | 3.0830% | 1.2673% | 0.1933% | 5.3705% | 3.3794% | 0.291% | 4.5820% | 2.2912% | 1.2135% | 3.9508% | 0.3376% | 2.2075% |
| 10 | 2.9229% | 1.1818% | 0.1929% | 5.0975% | 3.2936% | 0.290% | 4.6452% | 2.1543% | 1.1990% | 3.7548% | 0.3164% | 2.0834% |
| 65+ years old, Women, No medical history* | | | | | | | | | | | | |
| 1 | 4.8210% | 23.7758% | 1.1007% | 6.0826% | 13.663% | 2.162% | 4.1992% | 5.8818% | 6.6972% | 5.5596% | 12.0403% | 8.6640% |
| 2 | 5.0457% | 7.6141% | 1.0888% | 7.7640% | 9.7660% | 2.117% | 4.9466% | 4.5662% | 6.2768% | 6.4650% | 4.2190% | 6.4314% |
| 3 | 4.5270% | 5.5069% | 1.0770% | 7.3126% | 8.6814% | 2.073% | 5.2737% | 3.7764% | 5.9061% | 5.9484% | 3.1154% | 5.2219% |
| 4 | 4.1021% | 4.4628% | 1.0656% | 6.7734% | 8.0416% | 2.031% | 5.4977% | 3.2766% | 5.5767% | 5.4520% | 2.5606% | 4.4779% |
| 5 | 3.7631% | 3.8161% | 1.0543% | 6.2913% | 7.5957% | 1.990% | 5.6704% | 2.9225% | 5.2821% | 5.0339% | 2.2133% | 3.9600% |
| 6 | 3.4873% | 3.3678% | 1.0433% | 5.8761% | 7.2576% | 1.951% | 5.8117% | 2.6544% | 5.0171% | 4.6840% | 1.9705% | 3.5728% |
| 7 | 3.2580% | 3.0350% | 1.0326% | 5.5190% | 6.9876% | 1.914% | 5.9319% | 2.4423% | 4.7774% | 4.3880% | 1.7889% | 3.2693% |
| 8 | 3.0636% | 2.7760% | 1.0220% | 5.2094% | 6.7642% | 1.878% | 6.0367% | 2.2690% | 4.5596% | 4.1343% | 1.6468% | 3.0233% |
| 9 | 2.8964% | 2.5676% | 1.0117% | 4.9387% | 6.5745% | 1.844% | 6.1298% | 2.1240% | 4.3608% | 3.9141% | 1.5317% | 2.8189% |
| 10 | 2.7505% | 2.3955% | 1.0015% | 4.6998% | 6.4103% | 1.810% | 6.2136% | 2.0004% | 4.1786% | 3.7209% | 1.4362% | 2.6457% |
| 10-24 years old, Women, Medical history* | | | | | | | | | | | | |
| 1 | 5.9659% | 20.7115% | 0.2202% | 18.8707% | 10.728% | 0.390% | 8.2143% | 11.3933% | 0.7314% | 14.8347% | 1.1317% | 12.2033% |
| 2 | 5.9238% | 6.5458% | 0.2197% | 17.2486% | 7.6310% | 0.388% | 9.6395% | 7.5413% | 0.7261% | 13.1391% | 0.3817% | 8.3117% |
| 3 | 5.2129% | 4.7266% | 0.2193% | 14.4845% | 6.7745% | 0.387% | 10.2598% | 5.9359% | 0.7209% | 11.0005% | 0.2804% | 6.5748% |
| 4 | 4.6702% | 3.8275% | 0.2188% | 12.5860% | 6.2704% | 0.385% | 10.6833% | 5.0008% | 0.7157% | 9.5558% | 0.2299% | 5.5518% |
| 5 | 4.2508% | 3.2713% | 0.2183% | 11.2010% | 5.9196% | 0.384% | 11.0090% | 4.3693% | 0.7106% | 8.5078% | 0.1984% | 4.8573% |
| 6 | 3.9161% | 2.8860% | 0.2178% | 10.1384% | 5.6538% | 0.382% | 11.2753% | 3.9067% | 0.7056% | 7.7057% | 0.1764% | 4.3467% |
| 7 | 3.6415% | 2.6002% | 0.2174% | 9.2921% | 5.4417% | 0.381% | 11.5013% | 3.5495% | 0.7007% | 7.0676% | 0.1600% | 3.9517% |
| 8 | 3.4113% | 2.3779% | 0.2169% | 8.5990% | 5.2663% | 0.380% | 11.6982% | 3.2635% | 0.6958% | 6.5451% | 0.1472% | 3.6348% |
| 9 | 3.2146% | 2.1990% | 0.2164% | 8.0186% | 5.1174% | 0.378% | 11.8729% | 3.0281% | 0.6910% | 6.1078% | 0.1368% | 3.3736% |
| 10 | 3.0443% | 2.0513% | 0.2159% | 7.5241% | 4.9887% | 0.377% | 12.0302% | 2.8301% | 0.6862% | 5.7351% | 0.1283% | 3.1538% |
| 25-40 years old, Women, Medical history* | | | | | | | | | | | | |
| 1 | 6.5399% | 12.8968% | 0.4231% | 14.5391% | 5.0586% | 0.733% | 5.0985% | 9.3345% | 0.9237% | 11.0586% | 1.5540% | 8.2308% |
| 2 | 6.3450% | 3.9478% | 0.4213% | 14.4036% | 3.5660% | 0.728% | 6.0009% | 6.4912% | 0.9152% | 10.6613% | 0.5249% | 6.1874% |
| 3 | 5.5373% | 2.8397% | 0.4195% | 12.4010% | 3.1580% | 0.723% | 6.3953% | 5.1848% | 0.9069% | 9.1731% | 0.3856% | 5.0438% |
| 4 | 4.9369% | 2.2952% | 0.4178% | 10.9250% | 2.9188% | 0.718% | 6.6653% | 4.4062% | 0.8988% | 8.0921% | 0.3162% | 4.3353% |
| 5 | 4.4786% | 1.9594% | 0.4160% | 9.8127% | 2.7528% | 0.713% | 6.8733% | 3.8734% | 0.8908% | 7.2800% | 0.2729% | 3.8402% |
| 6 | 4.1156% | 1.7272% | 0.4143% | 8.9421% | 2.6272% | 0.708% | 7.0435% | 3.4793% | 0.8829% | 6.6448% | 0.2427% | 3.4690% |
| 7 | 3.8195% | 1.5552% | 0.4126% | 8.2391% | 2.5271% | 0.703% | 7.1881% | 3.1730% | 0.8752% | 6.1316% | 0.2201% | 3.1775% |
| 8 | 3.5721% | 1.4216% | 0.4109% | 7.6572% | 2.4444% | 0.698% | 7.3142% | 2.9263% | 0.8676% | 5.7064% | 0.2025% | 2.9409% |
| 9 | 3.3616% | 1.3142% | 0.4092% | 7.1660% | 2.3744% | 0.693% | 7.4262% | 2.7223% | 0.8601% | 5.3472% | 0.1883% | 2.7439% |
| 10 | 3.1798% | 1.2256% | 0.4076% | 6.7446% | 2.3138% | 0.688% | 7.5271% | 2.5501% | 0.8528% | 5.0387% | 0.1765% | 2.5769% |
| 40-65 years old, Women, Medical history* | | | | | | | | | | | | |
| 1 | 4.8748% | 14.6256% | 1.1965% | 7.0543% | 8.0245% | 0.906% | 3.2582% | 7.0539% | 3.1072% | 4.8156% | 5.5493% | 6.1627% |
| 2 | 5.0882% | 4.5078% | 1.1823% | 8.6413% | 5.6834% | 0.898% | 3.8415% | 5.2480% | 3.0136% | 5.8110% | 1.9000% | 4.9669% |
| 3 | 4.5606% | 3.2452% | 1.1685% | 8.0116% | 5.0396% | 0.890% | 4.0971% | 4.2808% | 2.9254% | 5.4240% | 1.3986% | 4.1416% |
| 4 | 4.1301% | 2.6240% | 1.1550% | 7.3552% | 4.6613% | 0.882% | 4.2723% | 3.6837% | 2.8423% | 5.0128% | 1.1477% | 3.6080% |
| 5 | 3.7872% | 2.2406% | 1.1418% | 6.7910% | 4.3984% | 0.874% | 4.4074% | 3.2667% | 2.7637% | 4.6546% | 0.9911% | 3.2261% |
| 6 | 3.5085% | 1.9755% | 1.1289% | 6.3151% | 4.1994% | 0.866% | 4.5180% | 2.9540% | 2.6894% | 4.3493% | 0.8817% | 2.9351% |
| 7 | 3.2770% | 1.7790% | 1.1163% | 5.9110% | 4.0407% | 0.859% | 4.6121% | 2.7083% | 2.6190% | 4.0879% | 0.8001% | 2.7038% |
| 8 | 3.0809% | 1.6263% | 1.1040% | 5.5640% | 3.9095% | 0.852% | 4.6942% | 2.5088% | 2.5521% | 3.8619% | 0.7362% | 2.5142% |
| 9 | 2.9122% | 1.5036% | 1.0919% | 5.2627% | 3.7982% | 0.844% | 4.7671% | 2.3427% | 2.4886% | 3.6645% | 0.6846% | 2.3553% |
| 10 | 2.7652% | 1.4023% | 1.0802% | 4.9983% | 3.7020% | 0.837% | 4.8328% | 2.2016% | 2.4282% | 3.4904% | 0.6417% | 2.2195% |
| 65+ years old, Women, Medical history* | | | | | | | | | | | | |
| 1 | 4.2785% | 27.5651% | 6.4120% | 5.7885% | 15.251% | 6.333% | 4.3692% | 6.1380% | 14.4543% | 4.7151% | 22.9415% | 9.6674% |
| 2 | 4.6087% | 8.9781% | 6.0256% | 7.4896% | 10.930% | 5.956% | 5.1460% | 4.7182% | 12.6289% | 5.7204% | 8.3839% | 6.9837% |
| 3 | 4.1803% | 6.5068% | 5.6832% | 7.0916% | 9.7234% | 5.621% | 5.4859% | 3.8895% | 11.2128% | 5.3507% | 6.2268% | 5.6227% |
| 4 | 3.8124% | 5.2786% | 5.3776% | 6.5885% | 9.0106% | 5.322% | 5.7187% | 3.3681% | 10.0823% | 4.9511% | 5.1329% | 4.7976% |
| 5 | 3.5129% | 4.5164% | 5.1031% | 6.1319% | 8.5136% | 5.053% | 5.8981% | 3.0001% | 9.1589% | 4.6011% | 4.4447% | 4.2280% |
| 6 | 3.2663% | 3.9876% | 4.8554% | 5.7358% | 8.1365% | 4.810% | 6.0449% | 2.7220% | 8.3904% | 4.3020% | 3.9621% | 3.8045% |
| 7 | 3.0596% | 3.5946% | 4.6305% | 5.3934% | 7.8352% | 4.589% | 6.1697% | 2.5024% | 7.7409% | 4.0455% | 3.6004% | 3.4740% |
| 8 | 2.8834% | 3.2888% | 4.4256% | 5.0956% | 7.5858% | 4.388% | 6.2786% | 2.3232% | 7.1848% | 3.8234% | 3.3167% | 3.2071% |
| 9 | 2.7310% | 3.0424% | 4.2380% | 4.8346% | 7.3740% | 4.203% | 6.3753% | 2.1735% | 6.7031% | 3.6292% | 3.0869% | 2.9858% |
| 10 | 2.5976% | 2.8390% | 4.0657% | 4.6038% | 7.1906% | 4.034% | 6.4624% | 2.0460% | 6.2821% | 3.4577% | 2.8959% | 2.7988% |
| 10-24 years old, Men, No medical history* | | | | | | | | | | | | |
| 1 | 6.1633% | 22.6175% | 0.0830% | 21.3689% | 12.308% | 0.272% | 8.4666% | 11.0104% | 0.6222% | 18.5790% | 1.0075% | 13.5911% |
| 2 | 6.0699% | 7.2068% | 0.0830% | 18.7873% | 8.7781% | 0.271% | 9.9332% | 7.3504% | 0.6184% | 15.3982% | 0.3397% | 9.0046% |
| 3 | 5.3257% | 5.2091% | 0.0829% | 15.5971% | 7.7984% | 0.270% | 10.5712% | 5.8000% | 0.6146% | 12.6355% | 0.2495% | 7.0660% |
| 4 | 4.7631% | 4.2202% | 0.0828% | 13.4675% | 7.2210% | 0.270% | 11.0068% | 4.8936% | 0.6108% | 10.8525% | 0.2045% | 5.9385% |
| 5 | 4.3303% | 3.6080% | 0.0828% | 11.9349% | 6.8190% | 0.269% | 11.3418% | 4.2801% | 0.6071% | 9.5886% | 0.1765% | 5.1785% |
| 6 | 3.9858% | 3.1837% | 0.0827% | 10.7690% | 6.5143% | 0.268% | 11.6155% | 3.8299% | 0.6035% | 8.6353% | 0.1570% | 4.6226% |
| 7 | 3.7037% | 2.8688% | 0.0826% | 9.8461% | 6.2710% | 0.267% | 11.8479% | 3.4820% | 0.5998% | 7.8849% | 0.1424% | 4.1941% |
| 8 | 3.4675% | 2.6239% | 0.0826% | 9.0935% | 6.0697% | 0.267% | 12.0503% | 3.2031% | 0.5963% | 7.2755% | 0.1310% | 3.8514% |
| 9 | 3.2660% | 2.4267% | 0.0825% | 8.4657% | 5.8989% | 0.266% | 12.2299% | 2.9733% | 0.5927% | 6.7686% | 0.1218% | 3.5697% |
| 10 | 3.0917% | 2.2639% | 0.0824% | 7.9324% | 5.7510% | 0.265% | 12.3915% | 2.7800% | 0.5892% | 6.3390% | 0.1141% | 3.3331% |
| 25-40 years old, Men, No medical history* | | | | | | | | | | | | |
| 1 | 6.7519% | 14.1487% | 0.1597% | 16.6667% | 5.8313% | 0.5119% | 5.2578% | 9.0036% | 0.7860% | 14.1550% | 1.3838% | 9.2801% |
| 2 | 6.4977% | 4.3525% | 0.1595% | 15.8328% | 4.1157% | 0.5092% | 6.1875% | 6.3165% | 0.7799% | 12.7100% | 0.4671% | 6.7725% |
| 3 | 5.6543% | 3.1327% | 0.1592% | 13.4525% | 3.6460% | 0.5067% | 6.5938% | 5.0589% | 0.7739% | 10.6869% | 0.3432% | 5.4698% |
| 4 | 5.0328% | 2.5328% | 0.1590% | 11.7652% | 3.3705% | 0.5041% | 6.8718% | 4.3061% | 0.7679% | 9.3058% | 0.2814% | 4.6758% |
| 5 | 4.5603% | 2.1626% | 0.1587% | 10.5160% | 3.1792% | 0.5016% | 7.0860% | 3.7896% | 0.7621% | 8.2988% | 0.2428% | 4.1260% |
| 6 | 4.1871% | 1.9066% | 0.1585% | 9.5487% | 3.0345% | 0.4991% | 7.2613% | 3.4070% | 0.7563% | 7.5255% | 0.2159% | 3.7164% |
| 7 | 3.8831% | 1.7169% | 0.1582% | 8.7735% | 2.9191% | 0.4966% | 7.4102% | 3.1091% | 0.7506% | 6.9089% | 0.1959% | 3.3962% |
| 8 | 3.6296% | 1.5695% | 0.1580% | 8.1354% | 2.8238% | 0.4942% | 7.5400% | 2.8690% | 0.7450% | 6.4031% | 0.1802% | 3.1373% |
| 9 | 3.4141% | 1.4510% | 0.1577% | 7.5991% | 2.7430% | 0.4917% | 7.6553% | 2.6702% | 0.7395% | 5.9792% | 0.1675% | 2.9225% |
| 10 | 3.2281% | 1.3532% | 0.1575% | 7.1408% | 2.6731% | 0.4893% | 7.7592% | 2.5024% | 0.7341% | 5.6174% | 0.1570% | 2.7407% |
| 40-65 years old, Men, No medical history | | | | | | | | | | | | |
| 1 | 5.0432% | 16.0293% | 0.4540% | 8.3367% | 9.2278% | 0.6323% | 3.3611% | 6.7864% | 2.6529% | 6.5144% | 4.9526% | 7.0072% |
| 2 | 5.2204% | 4.9685% | 0.4519% | 9.7398% | 6.5482% | 0.6284% | 3.9624% | 5.0953% | 2.5843% | 7.2658% | 1.6922% | 5.4774% |
| 3 | 4.6646% | 3.5793% | 0.4499% | 8.8727% | 5.8095% | 0.6244% | 4.2259% | 4.1685% | 2.5192% | 6.5809% | 1.2453% | 4.5214% |
| 4 | 4.2166% | 2.8951% | 0.4479% | 8.0658% | 5.3751% | 0.6206% | 4.4065% | 3.5933% | 2.4573% | 5.9774% | 1.0218% | 3.9153% |
| 5 | 3.8617% | 2.4726% | 0.4459% | 7.3980% | 5.0730% | 0.6167% | 4.5457% | 3.1905% | 2.3984% | 5.4851% | 0.8822% | 3.4863% |
| 6 | 3.5742% | 2.1804% | 0.4439% | 6.8462% | 4.8442% | 0.6130% | 4.6597% | 2.8877% | 2.3422% | 5.0806% | 0.7849% | 3.1617% |
| 7 | 3.3358% | 1.9637% | 0.4419% | 6.3839% | 4.6617% | 0.6092% | 4.7566% | 2.6496% | 2.2886% | 4.7424% | 0.7121% | 2.9051% |
| 8 | 3.1343% | 1.7953% | 0.4400% | 5.9909% | 4.5109% | 0.6055% | 4.8412% | 2.4559% | 2.2374% | 4.4552% | 0.6553% | 2.6958% |
| 9 | 2.9611% | 1.6599% | 0.4381% | 5.6521% | 4.3829% | 0.6019% | 4.9163% | 2.2944% | 2.1884% | 4.2076% | 0.6093% | 2.5209% |
| 10 | 2.8104% | 1.5481% | 0.4361% | 5.3565% | 4.2722% | 0.5983% | 4.9840% | 2.1573% | 2.1416% | 3.9916% | 0.5711% | 2.3719% |
| 65+ years old, Men, No medical history* | | | | | | | | | | | | |
| 1 | 4.4301% | 29.9732% | 2.5152% | 6.8932% | 17.430% | 4.4962% | 4.5063% | 5.8980% | 12.5557% | 6.3868% | 20.6943% | 5.8980% |
| 2 | 4.7323% | 9.8715% | 2.4535% | 8.4988% | 12.539% | 4.3027% | 5.3068% | 4.5758% | 11.1551% | 7.1610% | 7.4947% | 4.5758% |
| 3 | 4.2789% | 7.1640% | 2.3947% | 7.8988% | 11.166% | 4.1252% | 5.6570% | 3.7836% | 10.0356% | 6.4987% | 5.5594% | 3.7836% |
| 4 | 3.8949% | 5.8156% | 2.3387% | 7.2617% | 10.353% | 3.9618% | 5.8968% | 3.2824% | 9.1203% | 5.9094% | 4.5799% | 3.2824% |
| 5 | 3.5843% | 4.9779% | 2.2853% | 6.7108% | 9.7862% | 3.8108% | 6.0816% | 2.9275% | 8.3580% | 5.4268% | 3.9643% | 2.9275% |
| 6 | 3.3295% | 4.3963% | 2.2342% | 6.2447% | 9.3556% | 3.6709% | 6.2329% | 2.6587% | 7.7134% | 5.0294% | 3.5329% | 2.6587% |
| 7 | 3.1163% | 3.9640% | 2.1854% | 5.8482% | 9.0114% | 3.5409% | 6.3614% | 2.4461% | 7.1610% | 4.6968% | 3.2097% | 2.4461% |
| 8 | 2.9350% | 3.6272% | 2.1387% | 5.5073% | 8.7264% | 3.4198% | 6.4736% | 2.2724% | 6.6825% | 4.4139% | 2.9563% | 2.2724% |
| 9 | 2.7783% | 3.3560% | 2.0939% | 5.2109% | 8.4842% | 3.3067% | 6.5731% | 2.1271% | 6.2639% | 4.1699% | 2.7511% | 2.1271% |
| 10 | 2.6414% | 3.1319% | 2.0509% | 4.9506% | 8.2745% | 3.2009% | 6.6629% | 2.0033% | 5.8947% | 3.9568% | 2.5806% | 2.0033% |
| 10-24 years old, Men, Medical history* | | | | | | | | | | | | |
| 1 | 5.5022% | 26.2558% | 0.5090% | 20.6512% | 13.752% | 0.8268% | 8.8012% | 11.4199% | 1.4525% | 16.5082% | 2.0360% | 14.9663% |
| 2 | 5.5746% | 8.5012% | 0.5065% | 18.3517% | 9.8311% | 0.8201% | 10.3225% | 7.5545% | 1.4317% | 14.1692% | 0.6888% | 9.6717% |
| 3 | 4.9418% | 6.1567% | 0.5039% | 15.2830% | 8.7395% | 0.8134% | 10.9839% | 5.9452% | 1.4115% | 11.7491% | 0.5062% | 7.5360% |
| 4 | 4.4464% | 4.9928% | 0.5014% | 13.2189% | 8.0956% | 0.8068% | 11.4354% | 5.0082% | 1.3918% | 10.1508% | 0.4151% | 6.3071% |
| 5 | 4.0592% | 4.2709% | 0.4989% | 11.7281% | 7.6469% | 0.8004% | 11.7825% | 4.3755% | 1.3727% | 9.0044% | 0.3582% | 5.4839% |
| 6 | 3.7479% | 3.7703% | 0.4964% | 10.5915% | 7.3067% | 0.7940% | 12.0662% | 3.9120% | 1.3541% | 8.1332% | 0.3186% | 4.8844% |
| 7 | 3.4912% | 3.3984% | 0.4940% | 9.6902% | 7.0349% | 0.7878% | 12.3070% | 3.5542% | 1.3361% | 7.4438% | 0.2890% | 4.4238% |
| 8 | 3.2752% | 3.1089% | 0.4915% | 8.9544% | 6.8100% | 0.7816% | 12.5166% | 3.2677% | 1.3184% | 6.8815% | 0.2659% | 4.0564% |
| 9 | 3.0902% | 2.8759% | 0.4891% | 8.3400% | 6.6191% | 0.7755% | 12.7026% | 3.0318% | 1.3013% | 6.4123% | 0.2472% | 3.7550% |
| 10 | 2.9295% | 2.6834% | 0.4867% | 7.8176% | 6.4538% | 0.7696% | 12.8700% | 2.8336% | 1.2846% | 6.0135% | 0.2317% | 3.5024% |
| 25-40 years old, Men, Medical history* | | | | | | | | | | | | |
| 1 | 6.0413% | 16.5736% | 0.9753% | 16.0521% | 6.5435% | 1.5495% | 5.4694% | 9.3575% | 1.8308% | 12.4322% | 2.7910% | 10.3347% |
| 2 | 5.9798% | 5.1486% | 0.9659% | 15.4268% | 4.6236% | 1.5258% | 6.4352% | 6.5032% | 1.7979% | 11.5907% | 0.9466% | 7.3419% |
| 3 | 5.2561% | 3.7100% | 0.9566% | 13.1549% | 4.0972% | 1.5029% | 6.8572% | 5.1935% | 1.7661% | 9.8636% | 0.6959% | 5.8810% |
| 4 | 4.7059% | 3.0013% | 0.9476% | 11.5278% | 3.7883% | 1.4806% | 7.1459% | 4.4131% | 1.7355% | 8.6472% | 0.5707% | 5.0030% |
| 5 | 4.2813% | 2.5635% | 0.9387% | 10.3175% | 3.5737% | 1.4590% | 7.3683% | 3.8791% | 1.7059% | 7.7468% | 0.4926% | 4.3997% |
| 6 | 3.9429% | 2.2606% | 0.9300% | 9.3776% | 3.4113% | 1.4381% | 7.5502% | 3.4843% | 1.6773% | 7.0488% | 0.4382% | 3.9527% |
| 7 | 3.6654% | 2.0360% | 0.9214% | 8.6229% | 3.2819% | 1.4177% | 7.7049% | 3.1774% | 1.6496% | 6.4885% | 0.3975% | 3.6048% |
| 8 | 3.4328% | 1.8615% | 0.9130% | 8.0007% | 3.1749% | 1.3979% | 7.8396% | 2.9302% | 1.6228% | 6.0266% | 0.3657% | 3.3243% |
| 9 | 3.2344% | 1.7211% | 0.9047% | 7.4772% | 3.0842% | 1.3786% | 7.9593% | 2.7259% | 1.5969% | 5.6378% | 0.3400% | 3.0922% |
| 10 | 3.0625% | 1.6053% | 0.8966% | 7.0293% | 3.0058% | 1.3598% | 8.0671% | 2.5534% | 1.5718% | 5.3050% | 0.3187% | 2.8962% |
| 40-65 years old, Men, Medical history* | | | | | | | | | | | | |
| 1 | 4.4804% | 18.7399% | 2.7305% | 7.9614% | 10.332% | 1.910% | 3.4977% | 7.0725% | 6.0283% | 5.5554% | 9.8037% | 7.8644% |
| 2 | 4.7731% | 5.8738% | 2.6579% | 9.4246% | 7.3446% | 1.874% | 4.1230% | 5.2585% | 5.6855% | 6.4613% | 3.4075% | 5.9781% |
| 3 | 4.3112% | 4.2371% | 2.5891% | 8.6271% | 6.5191% | 1.839% | 4.3970% | 4.2886% | 5.3797% | 5.9455% | 2.5133% | 4.8904% |
| 4 | 3.9220% | 3.4295% | 2.5238% | 7.8638% | 6.0334% | 1.806% | 4.5846% | 3.6900% | 5.1050% | 5.4496% | 2.0647% | 4.2123% |
| 5 | 3.6077% | 2.9302% | 2.4617% | 7.2258% | 5.6954% | 1.774% | 4.7293% | 3.2720% | 4.8571% | 5.0318% | 1.7840% | 3.7367% |
| 6 | 3.3501% | 2.5846% | 2.4025% | 6.6957% | 5.4394% | 1.743% | 4.8478% | 2.9586% | 4.6321% | 4.6822% | 1.5879% | 3.3793% |
| 7 | 3.1349% | 2.3282% | 2.3462% | 6.2501% | 5.2351% | 1.713% | 4.9486% | 2.7124% | 4.4270% | 4.3864% | 1.4413% | 3.0981% |
| 8 | 2.9518% | 2.1289% | 2.2924% | 5.8702% | 5.0662% | 1.684% | 5.0365% | 2.5125% | 4.2393% | 4.1328% | 1.3266% | 2.8694% |
| 9 | 2.7938% | 1.9686% | 2.2410% | 5.5420% | 4.9229% | 1.656% | 5.1146% | 2.3460% | 4.0669% | 3.9128% | 1.2338% | 2.6790% |
| 10 | 2.6557% | 1.8362% | 2.1919% | 5.2553% | 4.7989% | 1.629% | 5.1849% | 2.2047% | 3.9080% | 3.7197% | 1.1567% | 2.5172% |
| 65+ years old, Men, Medical history* | | | | | | | | | | | | |
| 1 | 3.9238% | 34.5059% | 13.7055% | 6.5689% | 19.404% | 12.589% | 4.6884% | 6.1547% | 25.2613% | 5.4428% | 37.5614% | 12.0251% |
| 2 | 4.3144% | 11.6137% | 12.0535% | 8.2085% | 14.006% | 11.181% | 5.5203% | 4.7280% | 20.1669% | 6.3642% | 14.6364% | 8.2211% |
| 3 | 3.9446% | 8.4512% | 10.7569% | 7.6681% | 12.484% | 10.057% | 5.8842% | 3.8968% | 16.7824% | 5.8681% | 10.9695% | 6.5103% |
| 4 | 3.6143% | 6.8695% | 9.7122% | 7.0699% | 11.582% | 9.138% | 6.1333% | 3.3740% | 14.3707% | 5.3850% | 9.0837% | 5.5010% |
| 5 | 3.3412% | 5.8849% | 8.8524% | 6.5463% | 10.952% | 8.373% | 6.3252% | 3.0051% | 12.5650% | 4.9761% | 7.8883% | 4.8149% |
| 6 | 3.1143% | 5.2002% | 8.1325% | 6.1003% | 10.473% | 7.726% | 6.4824% | 2.7264% | 11.1624% | 4.6331% | 7.0458% | 4.3103% |
| 7 | 2.9228% | 4.6907% | 7.5209% | 5.7193% | 10.090% | 7.172% | 6.6159% | 2.5063% | 10.0415% | 4.3424% | 6.4121% | 3.9197% |
| 8 | 2.7589% | 4.2937% | 6.9948% | 5.3908% | 9.7735% | 6.692% | 6.7323% | 2.3267% | 9.1252% | 4.0929% | 5.9138% | 3.6062% |
| 9 | 2.6165% | 3.9737% | 6.5375% | 5.1045% | 9.5038% | 6.272% | 6.8358% | 2.1767% | 8.3622% | 3.8763% | 5.5092% | 3.3477% |
| 10 | 2.4916% | 3.7091% | 6.1364% | 4.8526% | 9.2702% | 5.902% | 6.9289% | 2.0489% | 7.7169% | 3.6860% | 5.1724% | 3.1301% |

*At diagnosis. Values are estimated from parametric survival modelling of the real-world 2014-2016 incident cohorts with details described in the main article and Supplementary Methods. Variations of 20% were used as lower and upper bounds. In probabilistic sensitivity analysis, uncertainties were modelled using Cholesky decomposition on the variance-covariance matrix for the underlying parameters generated in each regression analysis for all distributions. The probabilities were also validated by comparing simulated values with observed values (Supplementary Tables 4-7). Abbreviations: 2 – To, COM – New-onset comorbidities, DEAD – All-cause deaths, LOW – Low-intensity service users, NTRD – Non-treatment-resistant depression, TCOM – New-onset post-TRD comorbidities, tp – Transition probabilities, TRD – treatment-resistant depression.

**Supplementary Table 3**. Medical conditions to define baseline medical history and new-onset comorbidities

| **Diseases / Conditions** | **ICD-9-CM codes** | **Type of diagnoses** |
| --- | --- | --- |
| Myocardial Infarction | 410 | Physical history / comorbidity |
| Myocardial Infarction | 412 | Physical history / comorbidity |
| Congestive heart failure | 428 | Physical history / comorbidity |
| Peripheral vascular disease | 440 | Physical history / comorbidity |
| Peripheral vascular disease | 441 | Physical history / comorbidity |
| Peripheral vascular disease | 442 | Physical history / comorbidity |
| Peripheral vascular disease | 443 | Physical history / comorbidity |
| Peripheral vascular disease | 444 | Physical history / comorbidity |
| Peripheral vascular disease | 445 | Physical history / comorbidity |
| Peripheral vascular disease | 446 | Physical history / comorbidity |
| Peripheral vascular disease | 447 | Physical history / comorbidity |
| Peripheral vascular disease | 448 | Physical history / comorbidity |
| Peripheral vascular disease | 557 | Physical history / comorbidity |
| Peripheral vascular disease | 785.4 | Physical history / comorbidity |
| Cardiovascular disease | 430 | Physical history / comorbidity |
| Cardiovascular disease | 431 | Physical history / comorbidity |
| Cardiovascular disease | 432 | Physical history / comorbidity |
| Cardiovascular disease | 433 | Physical history / comorbidity |
| Cardiovascular disease | 434 | Physical history / comorbidity |
| Cardiovascular disease | 435 | Physical history / comorbidity |
| Cardiovascular disease | 436 | Physical history / comorbidity |
| Cardiovascular disease | 437 | Physical history / comorbidity |
| Cardiovascular disease | 438 | Physical history / comorbidity |
| Dementia | 290 | Physical history / comorbidity |
| Chronic obstructive pulmonary disease | 490 | Physical history / comorbidity |
| Chronic obstructive pulmonary disease | 491 | Physical history / comorbidity |
| Chronic obstructive pulmonary disease | 492 | Physical history / comorbidity |
| Chronic obstructive pulmonary disease | 493 | Physical history / comorbidity |
| Chronic obstructive pulmonary disease | 494 | Physical history / comorbidity |
| Chronic obstructive pulmonary disease | 495 | Physical history / comorbidity |
| Chronic obstructive pulmonary disease | 496 | Physical history / comorbidity |
| Chronic obstructive pulmonary disease | 500 | Physical history / comorbidity |
| Chronic obstructive pulmonary disease | 505 | Physical history / comorbidity |
| Chronic obstructive pulmonary disease | 506.4 | Physical history / comorbidity |
| Chronic obstructive pulmonary disease | 770.7 | Physical history / comorbidity |
| Connective tissue disease | 710 | Physical history / comorbidity |
| Connective tissue disease | 714 | Physical history / comorbidity |
| Connective tissue disease | 725 | Physical history / comorbidity |
| Pelvic ulcer disease | 531 | Physical history / comorbidity |
| Pelvic ulcer disease | 532 | Physical history / comorbidity |
| Pelvic ulcer disease | 533 | Physical history / comorbidity |
| Pelvic ulcer disease | 534 | Physical history / comorbidity |
| Liver diseases | 571 | Physical history / comorbidity |
| Liver diseases | 456 | Physical history / comorbidity |
| Liver diseases | 456.1 | Physical history / comorbidity |
| Liver diseases | 456.2 | Physical history / comorbidity |
| Liver diseases | 572.2 | Physical history / comorbidity |
| Liver diseases | 572.3 | Physical history / comorbidity |
| Liver diseases | 572.4 | Physical history / comorbidity |
| Liver diseases | 572.5 | Physical history / comorbidity |
| Liver diseases | 572.6 | Physical history / comorbidity |
| Liver diseases | 572.7 | Physical history / comorbidity |
| Liver diseases | 572.8 | Physical history / comorbidity |
| Diabetes mellitus | 250.0 | Physical history / comorbidity |
| Diabetes mellitus | 250.1 | Physical history / comorbidity |
| Diabetes mellitus | 250.2 | Physical history / comorbidity |
| Diabetes mellitus | 250.3 | Physical history / comorbidity |
| Diabetes mellitus | 250.4 | Physical history / comorbidity |
| Diabetes mellitus | 250.5 | Physical history / comorbidity |
| Diabetes mellitus | 250.6 | Physical history / comorbidity |
| Diabetes mellitus | 250.7 | Physical history / comorbidity |
| Hemiplegia | 342 | Physical history / comorbidity |
| Hemiplegia | 344.1 | Physical history / comorbidity |
| Chronic kidney disease | 582 | Physical history / comorbidity |
| Chronic kidney disease | 583 | Physical history / comorbidity |
| Chronic kidney disease | 585 | Physical history / comorbidity |
| Chronic kidney disease | 586 | Physical history / comorbidity |
| Chronic kidney disease | 588 | Physical history / comorbidity |
| Any tumors | 140 | Physical history / comorbidity |
| Any tumors | 141 | Physical history / comorbidity |
| Any tumors | 142 | Physical history / comorbidity |
| Any tumors | 143 | Physical history / comorbidity |
| Any tumors | 144 | Physical history / comorbidity |
| Any tumors | 145 | Physical history / comorbidity |
| Any tumors | 146 | Physical history / comorbidity |
| Any tumors | 147 | Physical history / comorbidity |
| Any tumors | 148 | Physical history / comorbidity |
| Any tumors | 149 | Physical history / comorbidity |
| Any tumors | 150 | Physical history / comorbidity |
| Any tumors | 151 | Physical history / comorbidity |
| Any tumors | 152 | Physical history / comorbidity |
| Any tumors | 153 | Physical history / comorbidity |
| Any tumors | 154 | Physical history / comorbidity |
| Any tumors | 155 | Physical history / comorbidity |
| Any tumors | 156 | Physical history / comorbidity |
| Any tumors | 157 | Physical history / comorbidity |
| Any tumors | 158 | Physical history / comorbidity |
| Any tumors | 159 | Physical history / comorbidity |
| Any tumors | 160 | Physical history / comorbidity |
| Any tumors | 161 | Physical history / comorbidity |
| Any tumors | 162 | Physical history / comorbidity |
| Any tumors | 163 | Physical history / comorbidity |
| Any tumors | 164 | Physical history / comorbidity |
| Any tumors | 165 | Physical history / comorbidity |
| Any tumors | 166 | Physical history / comorbidity |
| Any tumors | 167 | Physical history / comorbidity |
| Any tumors | 168 | Physical history / comorbidity |
| Any tumors | 169 | Physical history / comorbidity |
| Any tumors | 170 | Physical history / comorbidity |
| Any tumors | 171 | Physical history / comorbidity |
| Any tumors | 172 | Physical history / comorbidity |
| Any tumors | 174 | Physical history / comorbidity |
| Any tumors | 175 | Physical history / comorbidity |
| Any tumors | 176 | Physical history / comorbidity |
| Any tumors | 177 | Physical history / comorbidity |
| Any tumors | 178 | Physical history / comorbidity |
| Any tumors | 179 | Physical history / comorbidity |
| Any tumors | 180 | Physical history / comorbidity |
| Any tumors | 181 | Physical history / comorbidity |
| Any tumors | 182 | Physical history / comorbidity |
| Any tumors | 183 | Physical history / comorbidity |
| Any tumors | 184 | Physical history / comorbidity |
| Any tumors | 185 | Physical history / comorbidity |
| Any tumors | 186 | Physical history / comorbidity |
| Any tumors | 187 | Physical history / comorbidity |
| Any tumors | 188 | Physical history / comorbidity |
| Any tumors | 189 | Physical history / comorbidity |
| Any tumors | 190 | Physical history / comorbidity |
| Any tumors | 191 | Physical history / comorbidity |
| Any tumors | 192 | Physical history / comorbidity |
| Any tumors | 193 | Physical history / comorbidity |
| Any tumors | 194 | Physical history / comorbidity |
| Any tumors | 195 | Physical history / comorbidity |
| Any tumors | 196 | Physical history / comorbidity |
| Any tumors | 197 | Physical history / comorbidity |
| Any tumors | 198 | Physical history / comorbidity |
| Any tumors | 199 | Physical history / comorbidity |
| Leukemia | 203 | Physical history / comorbidity |
| Leukemia | 204 | Physical history / comorbidity |
| Leukemia | 205 | Physical history / comorbidity |
| Leukemia | 206 | Physical history / comorbidity |
| Leukemia | 207 | Physical history / comorbidity |
| Leukemia | 208 | Physical history / comorbidity |
| Lymphoma | 200 | Physical history / comorbidity |
| Lymphoma | 201 | Physical history / comorbidity |
| Lymphoma | 202 | Physical history / comorbidity |
| AIDS | 042 | Physical history / comorbidity |
| Suicidal ideation | E95 | Mental history / comorbidity |
| Suicidal ideation | E98 | Mental history / comorbidity |
| Attention-deficit hyperactivity disorder | 314 | Mental history / comorbidity |
| Autism | 299.0 | Mental history / comorbidity |
| Psychosis | 295 | Mental history / comorbidity |
| Psychosis | 297 | Mental history / comorbidity |
| Psychosis | 298 | Mental history / comorbidity |
| Epilepsy | 345 | Mental history / comorbidity |
| Anxiety disorder | 300.0 | Mental history / comorbidity |
| Anxiety disorder | 300.2 | Mental history / comorbidity |
| Anxiety disorder | 309.8 | Mental history / comorbidity |
| Anxiety disorder | 308.3 | Mental history / comorbidity |
| Anxiety disorder | 293.84 | Mental history / comorbidity |
| Personality disorder | 301 | Mental history / comorbidity |
| Substance use disorder | 292 | Mental history / comorbidity |
| Substance use disorder | 304 | Mental history / comorbidity |
| Substance use disorder | 305.1 | Mental history / comorbidity |
| Substance use disorder | 305.2 | Mental history / comorbidity |
| Substance use disorder | 305.3 | Mental history / comorbidity |
| Substance use disorder | 305.4 | Mental history / comorbidity |
| Substance use disorder | 305.5 | Mental history / comorbidity |
| Substance use disorder | 305.6 | Mental history / comorbidity |
| Substance use disorder | 305.7 | Mental history / comorbidity |
| Substance use disorder | 305.8 | Mental history / comorbidity |
| Substance use disorder | 305.9 | Mental history / comorbidity |
| Bipolar disorder | 296.0 | Mental history / comorbidity |
| Bipolar disorder | 296.1 | Mental history / comorbidity |
| Bipolar disorder | 296.4 | Mental history / comorbidity |
| Bipolar disorder | 296.5 | Mental history / comorbidity |
| Bipolar disorder | 296.6 | Mental history / comorbidity |
| Bipolar disorder | 296.7 | Mental history / comorbidity |
| Bipolar disorder | 296.8 | Mental history / comorbidity |
| Obsessive-compulsive disorder | 300.3 | Mental history / comorbidity |
| Eating disorder | 307.1 | Mental history / comorbidity |
| Eating disorder | 307.51 | Mental history / comorbidity |

**Supplementary Table 4**. Akaike and Bayesian Information Criterion values

|  | **Akaike Information Criterion (AIC) values** | | | | | **Bayesian Information Criterion (BIC) values** | | | | |
| --- | --- | --- | --- | --- | --- | --- | --- | --- | --- | --- |
| **Probabilities** | **Weibull** | **Exponential** | **Log-logistic** | **Lognormal** | **Gompertz** | **Weibull** | **Exponential** | **Log-logistic** | **Lognormal** | **Gompertz** |
| tpNTRD2TRD | 28855.85 | 28877.45 | 28775.16 | **28496.35** | - | 28912.78 | 28926.25 | 28832.09 | **28553.29** | - |
| tpNTRD2COM | **30121.59** | 40444.9 | 30338.4 | 30477.84 | - | **30178.53** | 40493.71 | 30395.33 | 30534.78 | - |
| tpNTRD2DEAD | 7057.058 | 7326.919 | **7047.97** | 7076.449 | 7150.185 | 7113.997 | 7375.725 | **7104.909** | 7133.388 | 7207.125 |
| tpNTRD2LOW | 46386.74 | 46412.92 | 46006.71 | **45377.34** | - | 46443.67 | 46461.72 | 46063.65 | **45434.28** | - |
| tpTRD2TCOM | **5792.64** | 5869.878 | 5802.747 | 5865.437 | - | **5837.442** | 5908.279 | 5847.549 | 5910.238 | - |
| tpTRD2DEAD | 1292.766 | 1292.766 | **1248.233** | 1257.962 | 1255.021 | -12928.84 | 1331.168 | **1293.034** | 1302.764 | 1299.822 |
| tpTRD2LOW | **5048.234** | 5057.688 | 5050.888 | 5089.678 | - | **5093.036** | 5096.09 | 5095.69 | 5134.48 | - |
| tpCOM2TRD | 6130.076 | 6177.292 | 6123.194 | **6122.318** | - | 6176.95 | 6217.47 | 6170.069 | **6169.192** | - |
| tpCOM2DEAD | 4548.375 | 4693.763 | **4547.577** | 4564.339 | 4607.401 | 4595.249 | 4733.941 | **4594.452** | 4611.214 | 4654.276 |
| tpCOM2LOW | 11238.96 | 11295.77 | 11168.26 | **11032.54** | - | 11285.52 | 11335.68 | 11214.82 | **11079.1** | - |
| tpTCOM2DEAD | **415.3417** | 512.3679 | 421.5892 | 434.4001 | 501.0216 | **448.1863** | 540.5204 | 454.4338 | 467.2447 | 533.8662 |
| tpTCOM2LOW | 755.3223 | 756.6534 | 754.2174 | **753.3696** | - | 788.1669 | **784.8059** | 787.062 | 786.2142 | - |

In the parametric survival modelling using the real-world 2014-2016 incident cohorts, selection of survival function was based on the lowest AIC and/or BIC values. Gompertz distribution were tested specifically for mortality-related outcomes. Abbreviations: 2 – To, COM – New-onset comorbidities, DEAD – All-cause deaths, LOW – Low-intensity service users, NTRD – Non-treatment-resistant depression, TCOM – New-onset post-TRD comorbidities, tp – Transition probabilities, TRD – treatment-resistant depression.

**Supplementary Table 5** – Modelled and observed cumulative numbers of deaths and low-intensity service users for validation of derived transition probabilities

|  | Cumulative all-cause deaths | | Cumulative low-intensity service users | |
| --- | --- | --- | --- | --- |
|  | Observed | Predicted | Observed | Predicted |
| Cycle 1 | 686 | 377 | 3728 | 2662 |
| Cycle 2 | 1014 | 805 | 5547 | 4925 |
| Cycle 3 | 1253 | 1153 | 6839 | 6622 |
| **Cycle 4** | **1470** | **1440** | **7983** | **7961** |
| Cycle 5* | 1630 | 1681 | 8916 | 9061 |
| Cycle 6* | 1717 | 1886 | 9430 | 9991 |
| Cycle 7* | 1744 | 2064 | 9578 | 10793 |
| Cycle 8** | 1744 | 2064 | 9578 | 10793 |
| Cycle 9** | 1744 | 2219 | 9578 | 11495 |
| Cycle 10** | 1744 | 2355 | 9578 | 12116 |

Parametric survival modelling was performed using the 2014-2016 incident cohorts, which were followed up to December 2020 to derive hazard function and transition probabilities between health states. “Observed” refers to the cumulative outcomes actually occurred during the follow-up of the reference cohort. “Predicted” refers to the simulated cumulative outcomes using the derived time-varying transition probabilities given the same number of initial patients for model entry. Comparison of two values allow the assessment of goodness-of-fit and confirm the survival function selection in the parametric survival modelling. Cycle 4 was chosen for validation of derived transition probabilities since it was the maximum follow-up period of the all reference cohorts (2014-2016) being followed up towards the end of 2020. *Cycles in which events may not be observed due to limited follow-up time. For example, the cycle 5 of patients diagnosed in 2016 is equivalent to the year 2021, which is after the end of our actual follow-up, i.e., December 2020. **Cycles in which no events could be observed in the reference cohort since the longest follow-up was seven years.

**Supplementary Table 6**. Mean absolute percentage errors of predicted deaths at cycle 4 in parametric survival modelling

| **Stratified by age group** | **Below 65** | **Above 65** | **Total** |
| --- | --- | --- | --- |
| Predicted deaths at Cycle 4 | 430 | 1010 | 1440 |
| Observed deaths at Cycle 4 | 432 | 1038 | 1470 |
| Numerical error | -2 | -28 | -30 |
| Relative error | -0.004163 | -0.0271435 | -0.02039 |
| Absolute relative error | 0.00416303 | 0.02714348 | 0.02039004 |
| MAPE | - | - | 1.57% |
| **Stratified by sex** | **Female** | **Male** | **Total** |
| Predicted deaths at Cycle 4 | 732 | 708 | 1440 |
| Observed deaths at Cycle 4 | 728 | 742 | 1470 |
| Numerical error | 4 | -34 | -30 |
| Relative error | 0.00605232 | -0.0463335 | -0.02039 |
| Absolute relative error | 0.00605232 | 0.04633349 | 0.02039004 |
| MAPE | - | - | 2.62% |
| **Stratified by baseline medical history** | **Yes** | **No** | **Total** |
| Predicted deaths at Cycle 4 | 1096 | 344 | 1440 |
| Observed deaths at Cycle 4 | 1133 | 337 | 1470 |
| Numerical error | -37 | 7 | -30 |
| Relative error | -0.0325806 | 0.02059474 | -0.02039 |
| Absolute relative error | 0.03258057 | 0.02059474 | 0.02039004 |
| MAPE | - | - | 2.66% |

Abbreviation: MAPE – Mean absolute percentage error.

**Supplementary Table 7**. Mean absolute percentage errors of predicted low-intensity service users at cycle 4 in parametric survival modelling

| **Stratified by age group** | **Below 65** | **Above 65** | **Total** |
| --- | --- | --- | --- |
| Predicted low-intensity service users at Cycle 4 | 6965 | 996 | 7961 |
| Observed low-intensity service users at Cycle 4 | 6976 | 1007 | 7983 |
| Numerical error | -11 | -11 | -22 |
| Relative error | -0.0016203 | -0.0104618 | -0.0027356 |
| Absolute relative error | 0.00162033 | 0.01046184 | 0.00273562 |
| MAPE | - | - | 0.60% |
| **Stratified by sex** | **Female** | **Male** | **Total** |
| Predicted low-intensity service users at Cycle 4 | 5670 | 2291 | 7961 |
| Observed low-intensity service users at Cycle 4 | 5662 | 2321 | 7983 |
| Numerical error | 8 | -30 | -22 |
| Relative error | 0.00145584 | -0.0129606 | -0.0027356 |
| Absolute relative error | 0.00145584 | 0.01296055 | 0.00273562 |
| MAPE | - | - | 0.72% |
| **Stratified by baseline medical history** | **Yes** | **No** | **Total** |
| Predicted low-intensity service users at Cycle 4 | 2265 | 5696 | 7961 |
| Observed low-intensity service users at Cycle 4 | 2251 | 5732 | 7983 |
| Numerical error | 14 | -36 | -22 |
| Relative error | 0.00642989 | -0.006335 | -0.0027356 |
| Absolute relative error | 0.00642989 | 0.00633499 | 0.00273562 |
| MAPE | - | - | 0.64% |

Abbreviation: MAPE – Mean absolute percentage error.

**Supplementary Table 8.** Non-subsidised unit costs of service types in the public healthcare system of Hong Kong, adapted from Hospital Authority website in 2023

| **Service setting** | **Sub-setting** | **Service type** | **Unit cost** |
| --- | --- | --- | --- |
| Inpatient | - | General ward | HK$5,100 |
|  |  | Psychiatric ward | HK$2,340 |
|  |  | Intensive care unit | HK$24,400 |
|  |  | High dependency ward | HK$13,650 |
| Accident & Emergency (AE) | - | Accident & Emergency | HK$1,230 |
| Outpatient | Specialist service | Outpatient clinic (Psychiatric) | HK$1,190 |
|  |  | Outpatient clinic (Non-psychiatric) | HK$1,190 |
|  |  | Allied health clinic | HK$1,190 |
|  | General service | Outpatient clinic | HK$445 |
|  |  | Family medicine | HK$1190 |
|  | Day hospital | Psychiatric day hospital | HK$1,260 |
|  |  | Geriatric day hospital | HK$1,960 |
|  |  | Rehabilitation day hospital | HK$1,320 |
|  | Community service | Nursing (General) | HK$535 |
|  |  | Nursing (Psychiatric) | HK$1,550 |
|  |  | Allied health | HK$1,730 |

Medical costs were estimated by multiplying the obtained utilisation data by the above service-specific unit costs as:

1) Inpatient cost = cumulative number of hospitalisation days per patient-year × ward charge per day.

2) Accident & Emergency cost = frequency of emergency room attendance per patient-year × unit emergency service costs

3) Outpatient cost = frequency of outpatient per patient-year × unit outpatient service costs (by specialty)

Fourteen service-specific costs were then aggregated into an overall cost.

**Supplementary Table 9**. Justification of adapted utility weights

| **Health state** | **NTRD** | **TRD** | **NTRD-comorbid** | **TRD-comorbid** | **Low-intensity service user** |
| --- | --- | --- | --- | --- | --- |
| **Utility** | 0.68 | 0.54 | 0.56 | 0.45 | 0.85 |
| **Source** | Rathod 2022 | Rathod 2022 | Johansson 2013; Zhou 2017;  IsHak 2018;  Moussavi 2007 | Johansson 2013; Zhou 2017;  IsHak 2018;  Moussavi 2007 | Sapin 2004 |
| **Value set** | United Kingdom | United Kingdom | (United Kingdom) | (United Kingdom) | United Kingdom |
| **Adjustment** | None | None | Assume 17% reduction of utility compared with that of the non-comorbid NTRD state | Assume 17% reduction of utility compared with that of the non-comorbid TRD state | None |
| **Justification** | Best alternative:  1) The only study that used EQ5D to directly compare QoL of TRD vs. non-TRD with reported mean utility value;  2) Alternatives were not chosen due to proxies of TRD vs. non-TRD (e.g. responders vs. non-responders of single intervention) or use of other QoL instruments. | Best alternative:  1) The only study that used EQ5D to directly compare QoL of TRD vs. non-TRD with reported mean utility value;  2) Alternatives were not chosen due to proxies of TRD vs. non-TRD (e.g. responders vs. non-responders of single intervention) or use of other QoL instruments. | Utility values of comorbid depression were generally 71-95% of depression alone (5-29% reduction) across literature | Utility values of comorbid depression were generally 71-95% of depression alone (5-29% reduction) across literature | Best alternative:  1) Study context was the closest to our disease journey in model;  2) Utility values of responders and non-responders in the same study are consistent with our chosen value of TRD vs. NTRD;  3) Same country value set as other chosen values;  4) Large sample size. |
| **Range 1** | 0.61 - 0.70 | 0.48 - 0.60 | NA | NA | 0.83 - 0.87 |
| **Range 2** | 0.60 - 0.72 | 0.42 - 0.58 | 0.50 - 0.60 | 0.35 - 0.48 | 0.52 - 0.88 |
| **Range for SA** | 0.60 - 0.72 | 0.42 - 0.60 | 0.50 - 0.60 | 0.35 - 0.48 | 0.52 - 0.88 |

Range 1 refers to the 95% confidence interval of the point estimate reported in the chosen article. Range 2 refers to the range of possible values reported from other unchosen articles. The overlapping scopes of ranges 1 and ranges 2 were selected and the lower and upper bounds for the utilities. Priority was given to articles with 1) similar context and disease course to our model, 2) representativeness of depression patient population, 3) HRQoL evaluation using EQ5D system, 4) reasonable statistical power and 5) value set consistent to that of other chosen articles.

Abbreviations: NA – Not applicable, NTRD – Non-treatment-resistant depression, QoL – Quality of life, SA – Sensitivity analysis, TRD – Treatment-resistant depression.

**Supplementary Table 10A.** Quality assessment for the selected HRQoL articles, cross-sectional studies

| **Quality assessment of cross-sectional studies using the Agency for Healthcare Research and Quality Scale** | | | | | | | | | | | | | |
| --- | --- | --- | --- | --- | --- | --- | --- | --- | --- | --- | --- | --- | --- |
| Studies | Q1 | Q2 | Q3 | Q4 | Q5 | Q6 | Q7 | Q8 | Q9 | Q10 | Q11 | Total | Risk of bias |
| Rathod 2022 | + | + | + | + | + | - | + | - | + | + | + | 9 | Low |
| Zhou 2017 | + | + | + | + | - | + | + | + | - | + | - | 8 | Low |
| Johansson 2013 | + | + | + | + | + | - | + | - | + | + | + | 9 | Low |
| Moussavi 2007 | + | + | - | + | + | + | + | + | - | + | - | 8 | Low |

Q1: Define source of information (survey, record, review).

Q2: List inclusion and exclusion criteria for exposed and unexposed subjects (cases and controls) or refer to previous publications.

Q3: Indicate time period used for identifying patients.

Q4: Indicate whether or not subjects were consecutive if not population-based.

Q5: Indicate if evaluators of subjective components of study were masked to other aspects of the status of the participants.

Q6: Describe any assessments undertaken for quality assurance purposes (e.g., test/retest of primary outcome measurements).

Q7: Explain any patient exclusions from analysis.

Q8: Describe how confounding was assessed and/or controlled.

Q9: If applicable, explain how missing data were handled in the analysis.

Q10: Summarize patient response rates and completeness of data collection.

Q11: Clarify what follow-up, if any, was expected and the percentage of patients for which incomplete data or follow-up was obtained.

A score of less than 3 indicates high bias risk, 4-7 indicates moderate bias risk, and 8 or greater indicates low bias risk.

Abbreviation: HRQoL – Health-related quality of life.

**Supplementary Table 10B.** Quality assessment for the selected HRQoL articles, cohort study

| Studies | | Sapin 2004 |
| --- | --- | --- |
| Selection | Representativeness of the exposed cohort | * |
|  | Selection of the non exposed cohort | * |
|  | Ascertainment of exposure | * |
|  | Demonstration that outcome of interest was not present at start of study | * |
| Comparability | Comparability of cohorts on the basis of the design or analysis | N/A (non-comparative design) |
| Outcome | Assessment of outcome | * |
|  | Was follow-up long enough for outcomes to occur | * |
|  | Adequacy of follow up of cohorts | * |
| Total score |  | 7 |
| Quality |  | Good |

Quality assessment was performed using the Newcastle-Ottawa Scale.

Good quality: 3 or 4 stars in selection domain AND 1 or 2 stars in comparability domain AND 2 or 3 stars in outcome/exposure domain.

Fair quality: 2 stars in selection domain AND 1 or 2 stars in comparability domain AND 2 or 3 stars in outcome/exposure domain.

Poor quality: 0 or 1 star in selection domain OR 0 stars in comparability domain OR 0 or 1 stars in outcome/exposure domain.

**Supplementary Table 10C.** Quality assessment for the selected HRQoL articles, Randomized controlled trial

| **Studies** | **Selection bias** | | **Performance bias** | **Detection bias** | **Attrition bias** | **Reporting bias** |
| --- | --- | --- | --- | --- | --- | --- |
| **IsHak 2018 (STAR*D trial)** | Random sequence generation | Allocation concealment | Blinding of participants and personnel | Blinding of outcome assessment | Incomplete outcome data | Selective reporting |
| **Support for judgement** | "Treatment assignment using randomization occurs via a blocked randomization scheme, stratified by CSs, and by the treatment options that are acceptable to the participant at that particular level." | "The CRC notifies the IVR system that the participant is exiting the current level and entering the next treatment level and presents the participant’s treatment preferences. The IVR system identifies the next treatment option in the appropriate stratum and immediately responds to the CRC." | "...if the RCT is placebo controlled, both patient and physician are unsure as to whether the patient is even receiving an active therapy. This may have a negative impact on response and remission rates for patients partici- pating in RCTs. STAR*D deals with this issue by making treatments known to both the patient and the treating physi- cian, as in normal clinical practice, ..." | "Assessments were conducted by treat ment-blinded raters at exit from each treatment level."  "The primary outcome is ... at entry and exit from each treatment level through telephone interviews by assessors masked to treatment assignments." | No detailed descriptions on losses to follow-up | All prespecified outcomes were reported. |
| **Risk of bias** | Low | Low | Moderate | Low | Unclear | Low |

Quality assessment was performed using the Cochrane Collaboration's tool for assessing risk of bias.

**Supplementary Table 11.** Baseline characteristics of patients newly diagnosed with incident depression between 2014 and 2016 at cohort entry

| **Characteristics** | **Before matching** | **After matching (b)** | | | |
| --- | --- | --- | --- | --- | --- |
|  | **All patients  (N = 25190)** | **Patients with TRD (N = 4448)** | **Patients without TRD (N = 16712)** | **p-value** | **SMD** |
| **Age (mean, SD)** | 48.63 (18.67) | 46.67 (17.67) | 46.27 (17.36) | 0.183 | 0.022 |
| **Age group (N, %)** |  | - | - | 0.076 | 0.044 |
| 10-24 | 2699 (10.7) | 500 (11.2) | 1951 (11.7) | - | - |
| 25-40 | 6260 (24.9) | 1228 (27.6) | 4602 (27.5) | - | - |
| 41-65 | 11567 (45.9) | 2040 (45.9) | 7850 (47.0) | - | - |
| 65+ | 4664 (18.5) | 680 (15.3) | 2309 (13.8) | - | - |
| **Female (N, %)** | 17972 (71.4) | 3321 (74.7) | 12554 (75.1) | 0.544 | 0.011 |
| **History of physical conditions (N, %) (a)** | 5503 (21.8) | 878 (19.7) | 2819 (16.9) | <0.001 | 0.074 |
| Diabetes mellitus | 1604 (6.4) | 243 (5.5) | 797 (4.8) | 0.062 | 0.032 |
| Cerebrovascular vascular disease | 1448 (5.7) | 210 (4.7) | 675 (4.0) | 0.048 | 0.033 |
| Chronic pulmonary disease | 1221 (4.8) | 219 (4.0) | 666 (4.0) | 0.006 | 0.045 |
| Any tumors | 1147 (4.6) | 157 (3.5) | 569 (3.4) | 0.718 | 0.007 |
| Ulcer disease | 617 (2.4) | 96 (2.2) | 294 (1.8) | 0.09 | 0.029 |
| Moderate/severe renal disease | 445 (1.8) | 69 (1.6) | 164 (1.0) | 0.002 | 0.051 |
| Congestive heart failure | 527 (2.1) | 65 (1.5) | 170 (1.0) | 0.015 | 0.04 |
| Peripheral vascular disease | 369 (1.5) | 69 (1.6) | 147 (0.9) | <0.001 | 0.061 |
| Liver diseases | 265 (1.1) | 47 (1.1) | 128 (0.8) | 0.07 | 0.031 |
| Connective tissue disease | 222 (0.9) | 38 (0.9) | 127 (0.8) | 0.589 | 0.011 |
| Hemiplegia | 307 (1.2) | 36 (0.8) | 147 (0.9) | 0.72 | 0.008 |
| Myocardial infarction | 278 (1.1) | 31 (0.7) | 108 (0.6) | 0.789 | 0.006 |
| Dementia | 126 (0.5) | 12 (0.3) | 45 (0.3) | 1 | <0.001 |
| Acquired Immunodeficiency Syndrome | 33 (0.1) | 9 (0.2) | 17 (0.1) | 0.144 | 0.026 |
| Lymphoma | 39 (0.2) | 5 (0.1) | 21 (0.1) | 1 | 0.004 |
| Leukemia | 30 (0.1) | 2 (0.0) | 12 (0.1) | 0.72 | 0.008 |
| **History of psychiatric conditions (N, %)** | 3615 (14.4) | 575 (12.9) | 2274 (13.6) | 0.248 | 0.020 |
| Suicidal ideation or attempt | 1515 (6.0) | 254 (5.7) | 929 (5.6) | 0.723 | 0.007 |
| Anxiety disorder | 1042 (4.1) | 185 (4.2) | 650 (3.9) | 0.437 | 0.014 |
| Psychosis | 716 (2.8) | 71 (1.6) | 484 (2.9) | <0.001 | 0.088 |
| Substance use disorder | 434 (1.7) | 73 (1.6) | 268 (1.6) | 0.913 | 0.003 |
| Epilepsy | 259 (1.0) | 30 (0.7) | 162 (1.0) | 0.079 | 0.033 |
| Personality disorder | 205 (0.8) | 38 (0.9) | 138 (0.8) | 0.925 | 0.003 |
| Bipolar disorder | 101 (0.4) | 8 (0.2) | 66 (0.4) | 0.044 | 0.04 |
| Obsessive-Compulsive Disorder | 62 (0.2) | 10 (0.2) | 45 (0.3) | 0.725 | 0.009 |
| Attention-deficit Hyperactivity Disorder | 41 (0.2) | 3 (0.1) | 29 (0.2) | 0.161 | 0.031 |
| Eating disorder | 33 (0.1) | 8 (0.2) | 25 (0.1) | 0.81 | 0.007 |
| Autism | 12 (0.0) | 2 (0.0) | 8 (0.0) | 1 | 0.001 |
| **Follow-up period (mean years, SD)** | 5.18 (1.35) | 3.44 (1.70) | 3.48 (1.66) | 0.116 | 0.026 |

a. Categorization was based on the disease types included in the Charlson Comorbidity index.

b. In the cost analysis, 4030 patients were excluded from the overall incident due to matching process.

Abbreviation: SD − Standard deviation, SMD − Standardised mean difference, TRD − Treatment-resistant depression.

**Supplementary Table 12.** Causes of death in the 2014-2016 incident cohorts

| **Causes of death** | **Number of patients** | **Percentage** |
| --- | --- | --- |
| All-cause | 2308 | 100% |
| Diseases of the respiratory system | 802 | 34.7% |
| Neoplasm | 436 | 18.9% |
| Diseases of the circulatory system | 294 | 12.7% |
| External causes of death | 171 | 7.4% |
| Diseases of the genitourinary system | 102 | 4.4% |
| Diseases of the digestive system | 91 | 3.9% |
| Certain infectious and parasitic diseases | 78 | 3.4% |
| Unknown or not recorded | 242 | 10.5% |
| Undiagnosed | 26 | 1.1% |
| Other causes* | 66 | 2.9% |

The 2014-2016 reference incident cohorts contained 25,190 patients newly diagnosed between 2014 and 2016. The above table shows the distribution of causes of death by December 2020. *Other causes include diseases of the nervous system, skin, musculoskeletal system, blood-forming processes and immune mechanisms, organic including symptomatic and mental disorders (excluding depression), endocrine and metabolic diseases and congenital diseases.

**Supplementary Table 13A.** Undiscounted and discounted projected outcomes, base-case scenario (closed cohort model)

| **Study year** | **Calendar year equivalent** | **All-cause HRU cost** | **All-cause HRU cost (discounted)** | **Psychiatric HRU cost** | **Psychiatric HRU cost (discounted)** | **Life-years** | **QALYs** | **QALYs (discounted)** |
| --- | --- | --- | --- | --- | --- | --- | --- | --- |
| No residual pandemic effect in 2023 | | | | | | | | |
| 1 | 2023 | 309,876,580 | 309,876,580 | 51,165,597 | 51,165,597 | 9,010 | 6,034 | 6,034 |
| 2 | 2024 | 291,318,547 | 284,213,216 | 50,406,294 | 49,176,872 | 8,855 | 6,009 | 5,863 |
| 3 | 2025 | 272,792,718 | 259,648,036 | 49,116,413 | 46,749,709 | 8,728 | 6,001 | 5,712 |
| 4 | 2026 | 256,603,383 | 238,281,751 | 47,746,387 | 44,337,267 | 8,624 | 6,000 | 5,572 |
| 5 | 2027 | 242,566,093 | 219,752,909 | 46,387,272 | 42,024,579 | 8,537 | 6,004 | 5,440 |
| 6 | 2028 | 230,268,687 | 203,523,966 | 45,063,406 | 39,829,485 | 8,462 | 6,012 | 5,314 |
| 7 | 2029 | 219,359,577 | 189,153,076 | 43,782,311 | 37,753,350 | 8,398 | 6,021 | 5,192 |
| 8 | 2030 | 209,571,363 | 176,305,102 | 42,546,461 | 35,792,858 | 8,341 | 6,032 | 5,075 |
| 9 | 2031 | 200,704,312 | 164,727,376 | 41,356,548 | 33,943,245 | 8,292 | 6,044 | 4,960 |
| 10 | 2032 | 192,608,076 | 154,226,749 | 40,212,489 | 32,199,281 | 8,248 | 6,056 | 4,849 |
| **Cumulative value** | | 2,425,669,337 | 2,199,708,761 | 457,783,177 | 412,972,241 | 85,495 | 60,213 | 54,009 |
| With residual pandemic effect in 2023 | | | | | | | | |
| 1 | 2023 | 386,140,267 | 386,140,267 | 63,757,956 | 63,757,956 | 11,228 | 7,519 | 7,519 |
| 2 | 2024 | 363,014,918 | 354,160,896 | 62,811,780 | 61,279,786 | 11,034 | 7,488 | 7,305 |
| 3 | 2025 | 339,929,700 | 323,549,982 | 61,204,447 | 58,255,274 | 10,876 | 7,478 | 7,117 |
| 4 | 2026 | 319,756,011 | 296,925,243 | 59,497,244 | 55,249,106 | 10,747 | 7,477 | 6,943 |
| 5 | 2027 | 302,264,005 | 273,836,270 | 57,803,637 | 52,367,243 | 10,638 | 7,482 | 6,779 |
| 6 | 2028 | 286,940,086 | 253,613,225 | 56,153,955 | 49,631,914 | 10,545 | 7,491 | 6,621 |
| 7 | 2029 | 273,346,136 | 235,705,517 | 54,557,570 | 47,044,822 | 10,464 | 7,503 | 6,470 |
| 8 | 2030 | 261,148,946 | 219,695,529 | 53,017,565 | 44,601,834 | 10,394 | 7,517 | 6,323 |
| 9 | 2031 | 250,099,626 | 205,268,410 | 51,534,803 | 42,297,013 | 10,332 | 7,531 | 6,181 |
| 10 | 2032 | 240,010,826 | 192,183,475 | 50,109,180 | 40,123,842 | 10,278 | 7,546 | 6,042 |
| **Cumulative value** | | 3,022,650,521 | 2,741,078,816 | 570,448,139 | 514,608,789 | 106,536 | 75,032 | 67,301 |

Abbreviations: HRU – Healthcare resource utilization, QALY – Quality-adjusted life years.

**Supplementary Table 13B.** Undiscounted and discounted projected outcomes, scenario analysis (open cohort model)

| **Study year** | **Calendar year equivalent** | **All-cause HRU cost** | **All-cause HRU cost (discounted)** | **Psychiatric HRU cost** | **Psychiatric HRU cost (discounted)** | **Life-years** | **QALYs** | **QALYs (discounted)** |
| --- | --- | --- | --- | --- | --- | --- | --- | --- |
| No residual pandemic effect in 2023 | | | | | | | | |
| 1 | 2023 | 2,588,421,187 | 2,358,584,595 | 486,321,725 | 440,958,350 | 90,421 | 63,548 | 57,307 |
| 2 | 2024 | 2,591,282,967 | 2,355,973,342 | 488,139,501 | 441,669,882 | 90,884 | 63,931 | 57,539 |
| 3 | 2025 | 2,596,521,161 | 2,356,325,115 | 490,079,214 | 442,585,571 | 91,378 | 64,329 | 57,790 |
| 4 | 2026 | 2,611,883,704 | 2,365,491,248 | 493,871,000 | 445,050,937 | 92,259 | 65,007 | 58,266 |
| 5 | 2027 | 2,625,175,121 | 2,373,605,723 | 497,032,790 | 447,082,043 | 93,012 | 65,586 | 58,666 |
| 6 | 2028 | 2,643,760,090 | 2,386,242,185 | 501,188,860 | 449,933,068 | 93,976 | 66,319 | 59,184 |
| 7 | 2029 | 2,598,423,125 | 2,349,205,079 | 491,752,149 | 442,191,026 | 92,093 | 64,946 | 58,055 |
| 8 | 2030 | 2,572,264,776 | 2,328,588,216 | 486,150,232 | 437,726,664 | 90,955 | 64,109 | 57,381 |
| 9 | 2031 | 2,548,641,879 | 2,310,873,467 | 480,954,417 | 433,764,823 | 89,861 | 63,294 | 56,750 |
| 10 | 2032 | 2,530,013,809 | 2,297,889,964 | 476,704,471 | 430,712,370 | 88,925 | 62,588 | 56,229 |
| **Cumulative value** | | 25,906,387,821 | 23,482,778,932 | 4,892,194,358 | 4,411,674,735 | 913,762 | 643,657 | 577,166 |
| With residual pandemic effect in 2023 | | | | | | | | |
| 1 | 2023 | 2,664,673,044 | 2,434,836,725 | 498,912,031 | 453,548,701 | 92,638 | 65,033 | 58,792 |
| 2 | 2024 | 2,662,966,538 | 2,425,908,739 | 500,542,705 | 453,770,604 | 93,062 | 65,409 | 58,981 |
| 3 | 2025 | 2,663,655,566 | 2,420,225,251 | 502,166,589 | 454,090,617 | 93,526 | 65,806 | 59,196 |
| 4 | 2026 | 2,675,036,158 | 2,424,135,334 | 505,621,618 | 455,962,685 | 94,381 | 66,483 | 59,637 |
| 5 | 2027 | 2,684,858,636 | 2,427,675,406 | 508,446,603 | 457,422,300 | 95,112 | 67,064 | 60,004 |
| 6 | 2028 | 2,700,427,862 | 2,436,328,821 | 512,278,557 | 459,734,844 | 96,058 | 67,799 | 60,492 |
| 7 | 2029 | 2,652,398,775 | 2,395,749,127 | 502,525,052 | 451,480,667 | 94,160 | 66,428 | 59,332 |
| 8 | 2030 | 2,623,824,697 | 2,371,961,506 | 496,618,312 | 446,532,727 | 93,007 | 65,593 | 58,630 |
| 9 | 2031 | 2,598,022,627 | 2,351,400,919 | 491,130,025 | 442,116,142 | 91,901 | 64,781 | 57,971 |
| 10 | 2032 | 2,577,392,480 | 2,335,822,735 | 486,597,113 | 438,632,893 | 90,954 | 64,078 | 57,421 |
| **Cumulative value** | | 26,503,256,382 | 24,024,044,563 | 5,004,838,605 | 4,513,292,180 | 934,799 | 658,474 | 590,456 |

Abbreviations: HRU – Healthcare resource utilization, QALY – Quality-adjusted life years.

**Supplementary Figure 1**. Schematic presentation of scope of included patients in the closed and open cohort models

**
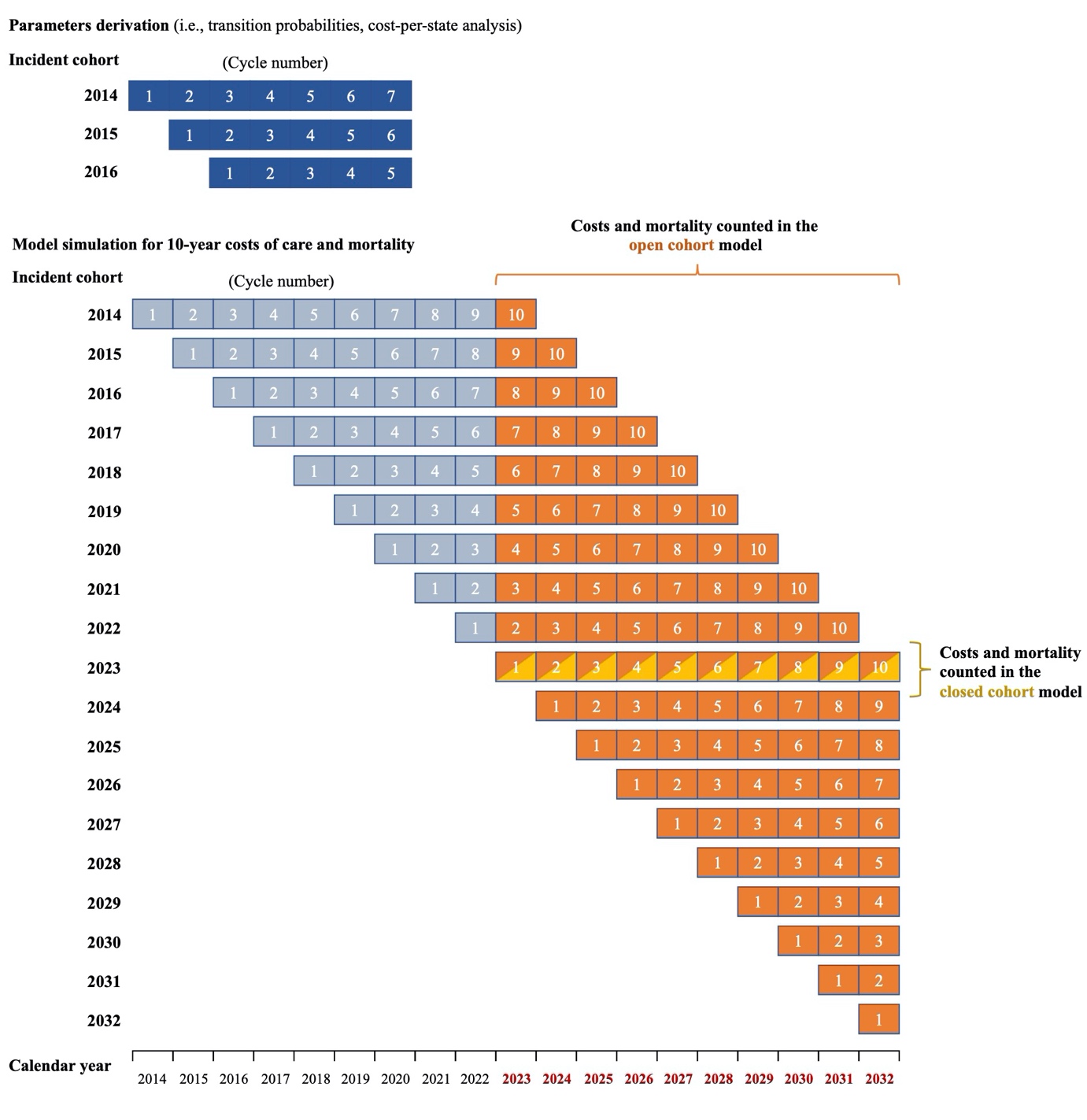
**

**Supplementary Figure 2.** Projected annual numbers of all-cause death from 2023 to 2032


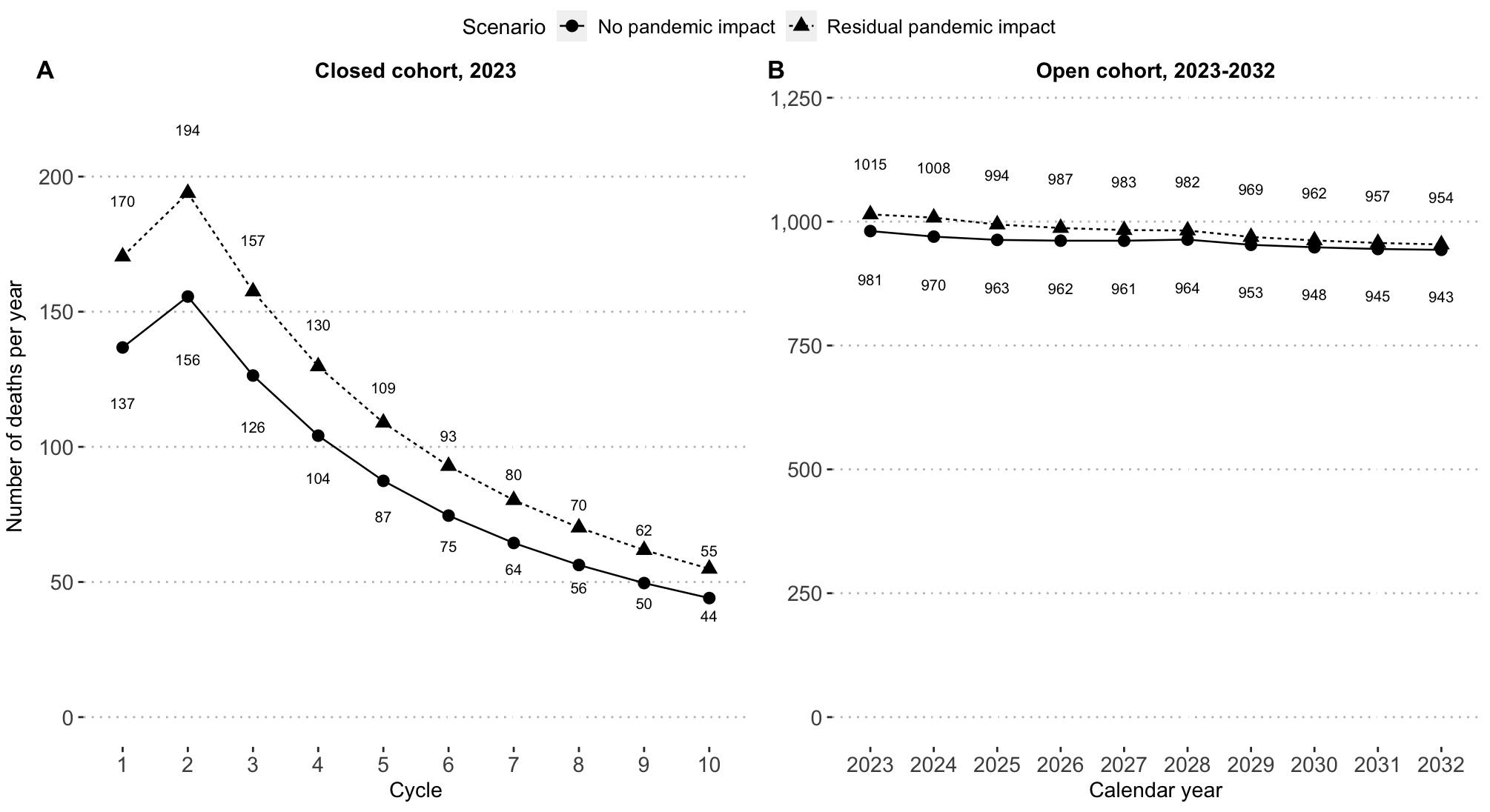


The closed cohort setting shows the projected annual new all-cause deaths among the incident patients diagnosed in 2023, with the tenth cycle equivalent to the year 2032. The initial number of patients at risk was 8,265. The open cohort setting shows the projected annual new all-cause deaths among the patients diagnosed in the recent 10 years counting from the corresponding calendar year.

**Supplementary Methods.** Detailed description on derivation of model input parameters

**Time-varying transition probabilities**

Modelling studies using Markov structure commonly assume fixed transition probabilities between health states for all cycles for simplicity. In chronic disease modelling, however, transition probabilities could vary over the prolonged disease course. For instance, we may anticipate that the probabilities of dying or developing treatment-resistant depression (TRD) from the non-TRD state to drop over time because, as cycles elapse, patients who remained as non-treatment-resistant were likely to exhibit long-term stability. Constant transition probabilities may introduce bias in unpredictable directions, we therefore used time-inhomogeneous transitions in our Markov model based on parametric survival modelling.

We first retrospectively identified a real-world cohort within our territory-wide electronic medical record (EMR) database, which was the patients who were newly diagnosed with depression between January 2014 and December 2016. We then followed up these patients until December 2020 to examine their transitions between health states by assessing whether they fulfilled our definition of the health states and the dates of entering the health states. Then, we conducted 12 parametric survival analyses to model the 12 transitions between the defined health states, censoring at non-target states or the end of 2020. For instance, the transition from Non-TRD to TRD was modelled by survival analysis among the non-TRD patients with the survival outcome being TRD, and patients were censored if they transited to the states other than TRD (e.g. new-onset comorbidities) earlier than becoming TRD, or did not develop TRD by the end of 2020. The survival models adjusted for age, sex and baseline medical history at diagnosis, therefore we were able to derive the probabilities stratified by subgroup at the later stage.

Given abundant choices of distributions for survival modelling, we fitted each transition using five recommended standard distributions (exponential, Weibull, lognormal, log-logistic and Gompertz), and we selected the best distribution based on the lowest Akaike information criterion (AIC) and Bayesian information criterion (BIC) values alongside visual inspection of the fitted models against Kaplan-Meier curves. We then obtained the regression ancillary parameters which were useful for the conversion into time-varying transition probabilities, and the estimates stratified by the subgroups were derived by simply substituting the right age groups, sex, and baseline medical history into the equations. Then, at cycle *t*, time-varying probabilities were converted using the formula P(t)=1-S(t)/S(t-1), where S(t) is the survival function of the distribution in the subgroups. Since uncertainties could be involved in the choices of survival distribution and goodness-of-fit, we performed validation on the derived transition probabilities. We compared the observed number of cumulative deaths and low-intensity service users that actually occurred in the 4^th^ year of follow-up among the 2014-2016 reference cohorts, to those “predicted” number of cumulative deaths and low-intensity service users which were estimated by Markov model simulation using our derived transition probabilities given the same number of initial patients for model entry. Cycle 4 was chosen for validation since it was the minimum follow-up period of the reference cohort, given that not all patients had follow-up of 5 years or beyond. The mean absolute percentage errors ranged from 0.6% to 2.66%, which indicated a highly accurate forecast.

**Costs of care**

The estimated of costs of care of each health states was performed using an epidemiological study design. The method was also consistent and described in detail in our previous cost analysis of TRD. Similar as the derivation of transition probabilities, we first retrospectively identified a real-world cohort within our territory-wide electronic medical record (EMR) database, which was the patients who were newly diagnosed with depression between January 2014 and December 2016. Then, we followed up their prescription records until December 2020 to identify whether they developed TRD, which was defined as having taken at least two antidepressant regimens for an adequate duration and had the third regimen to confirm refractoriness in the first two regimens throughout the follow-up. An adequate duration refers to the same antidepressant or combination regimen of at least 28 days with gaps no longer than 14 days within regimens. The date on which the TRD patients received the third regimen was defined as the index date. Patients who did not receive the third regimen by the end of follow-up were considered as non-TRD.

Then, we matched the TRD patients in the 2014-2016 incident cohorts 1:4 to the non-TRD patients on a propensity score estimated by age, sex, and baseline medical history before diagnosis. Since patients in the non-TRD group naturally did not have the third regimen, their index dates followed the ones of their matched TRD control within the same strata. We then followed up from index dates to death or end of study to examine the cumulative number of service attendance episodes and inpatient length of stay in bed-days across the outpatient, inpatient and emergency settings. Costs were then calculated in a bottom-up approach. We calculated the costs as the products of episodes or bed-days in 14 service types and service-specific unit costs released by the Hospital Authority, which were then aggregated as the overall cost. All-cause care indicated all types of care regardless of psychiatric or non-psychiatric resources. Psychiatric care included inpatient psychiatric ward, community psychiatric outreach, specialist outpatient clinic in psychiatry and psychiatric day hospital. We then fitted negative binomial regression to model the costs of care, with covariates being the matched variables (age group, sex, and baseline medical history), TRD status and new-onset comorbidities from index date to end of follow-up. Costs of care per state specific to 16 subgroups were obtained by substituting the desired patient characteristics and defining TRD and new-onset comorbidities in the regression equation. The states of low-intensity service user and all-cause death were cost-free since they were assumed to be not disease-related. Costs of drug treatment and psychotherapy were not included owing to incomplete data.

**Utilities**

Given the lack of quality-of-life measures within the EMR database, we obtained utility weight per state based on literature review on the health-related quality-of-life (HRQoL) of patients with TRD and comorbidities. We performed a comprehensive search using keywords ("quality of life"[Title/Abstract] AND "treatment-resistant depression"[Title/Abstract]) on PubMed and ("quality of life"[Title/Abstract] AND "depress*"[Title/Abstract] AND "comorbid*"[Title/Abstract]) on PubMed and Google search engines until 31 October 2022.

We then screened for relevant articles and extracted article data into an Excel file. The extracted data included publication year, study design, studied intervention if any, country, population, sample size, HRQoL instrument, and the results. Priority was given to articles with 1) similar context and disease course to our model, 2) representativeness of depression patient population, 3) HRQoL evaluation using EQ5D system, 4) reasonable statistical power and 5) value set consistent to that of other chosen articles. Given the scant HRQoL literature on the two comorbidity states specific to treatment resistance (NTRD-comorbid and TRD-comorbid), we assumed the utilities were 17% lower than those of the non-comorbid states of NTRD and TRD since utility values of comorbid depression are generally 71-95% of depression alone (5-29% reduction) across literature. Finally, quality assessment was done using the Agency for Healthcare Research and Quality Scale for cross-sectional studies, Newcastle-Ottawa Scale for cohort studies, and Cochrane Collaboration’s tool for risk of bias for randomized control trials. All selected articles passed our quality assessment satisfactorily.

**Number of newly diagnosed patients**

We first computed the annual incidences of depression between 2014 and 2018, which were the number of newly diagnosed patients in each year divided by the mid-year population of the year, then calculated the mean annual incidence. We then projected the number of new patients between 2019 and 2032 by multiplying the constant mean annual incidence by the official population projections released by the government. The process was performed in an age-specific manner using 5-year age bands. Given the occurrence of the unprecedented social movement in 2019 and COVID-19 pandemic from 2020 to 2022, a previous large-scale local prospective cohort study reported an increase in the prevalence of probable depression specific to the two events. We therefore assumed 1.72- and 1.53-fold increase in new cases between 2019 and 2022, with 52.5% of excess cases who eventually sought medical help and entered the public healthcare system.

Although the WHO has ended the COVID-19 global health emergency declaration in May 2023, slow economic recovery due to residual pandemic stress might persist. We therefore estimated two scenarios of new case number for 2023 by assuming 1) no pandemic from 2023 onwards and 2) residual pandemic impact in 2023 which resolved from 2024 onwards.
